# Wnt signalling is a bi-directional vulnerability of cancer cells

**DOI:** 10.18632/oncotarget.11203

**Published:** 2016-08-11

**Authors:** David J. Duffy, Aleksandar Krstic, Thomas Schwarzl, Melinda Halasz, Kristiina Iljin, Dirk Fey, Bridget Haley, Jenny Whilde, Saija Haapa-Paananen, Vidal Fey, Matthias Fischer, Frank Westermann, Kai-Oliver Henrich, Steffen Bannert, Desmond G. Higgins, Walter Kolch

**Affiliations:** ^1^ Systems Biology Ireland, University College Dublin, Belfield, Dublin, Ireland; ^2^ Conway Institute of Biomolecular and Biomedical Research, University College Dublin, Belfield, Dublin, Ireland; ^3^ School of Medicine, University College Dublin, Belfield, Dublin, Ireland; ^4^ Division of NB Genomics, German Cancer Research Center (DKFZ), Heidelberg, Germany; ^5^ Department of Paediatric Haematology and Oncology and Center for Molecular Medicine Cologne (CMMC), University Hospital Cologne, Cologne, Germany; ^6^ VTT Technical Research Centre of Finland, Espoo, Finland; ^7^ Current address: The Whitney Laboratory for Marine Bioscience, University of Florida, St. Augustine, Florida, USA; ^8^ Current address: European Molecular Biology Laboratory (EMBL), Heidelberg, Germany

**Keywords:** neuroblastoma, melanoma, colorectal cancer, MYC (c-MYC), mRNA sequencing (mRNA-seq)

## Abstract

Wnt signalling is involved in the formation, metastasis and relapse of a wide array of cancers. However, there is ongoing debate as to whether activation or inhibition of the pathway holds the most promise as a therapeutic treatment for cancer, with conflicting evidence from a variety of tumour types. We show that Wnt/β-catenin signalling is a bi-directional vulnerability of neuroblastoma, malignant melanoma and colorectal cancer, with hyper-activation or repression of the pathway both representing a promising therapeutic strategy, even within the same cancer type. Hyper-activation directs cancer cells to undergo apoptosis, even in cells oncogenically driven by β-catenin. Wnt inhibition blocks proliferation of cancer cells and promotes neuroblastoma differentiation. Wnt and retinoic acid co-treatments synergise, representing a promising combination treatment for MYCN-amplified neuroblastoma. Additionally, we report novel cross-talks between MYCN and β-catenin signalling, which repress normal β-catenin mediated transcriptional regulation. A β-catenin target gene signature could predict patient outcome, as could the expression level of its DNA binding partners, the TCF/LEFs. This β-catenin signature provides a tool to identify neuroblastoma patients likely to benefit from Wnt-directed therapy. Taken together, we show that Wnt/β-catenin signalling is a bi-directional vulnerability of a number of cancer entities, and potentially a more broadly conserved feature of malignant cells.

## INTRODUCTION

Wnt/β-catenin signalling is a key developmental pathway which is dysregulated in a wide variety of cancers [1–7]. Activation of this pathway can conversely lead to oncogenic or tumour suppressive effects in different cancer entities [8–16]. Indeed, in some tumours such as malignant melanoma the role of β-catenin activation is controversial, being linked to both pro- and anti-tumour effects [2, 10–14, 17, 18]. Similarly, in neuroblastoma contradictory evidence is emerging about the role played by Wnt/β-catenin signalling with both pathway activation and inhibition being mooted as potential therapeutic strategies, and β-catenin activation being linked to chemoresistance [1, 19–25]. Such opposing evidence makes it unclear whether the targeting of this pathway would lead to improvements or be detrimental for patients, potentially increasing their tumour burdens [26, 27]. However, decades of clinical experience shows that there is no enhanced cancer risk in patients taking lithium for bipolar disorder [28], despite lithium activating Wnt signalling through GSK3 inhibition [1, 29]. Rather, cancer risk was significantly lower in the lithium treated groups than the general population [28]. That universally inhibiting canonical Wnt signalling in cancers would be beneficial has also recently been questioned, as elevated Wnt/β-catenin signalling does not correlate with reduced patient survival in all types of cancer [4, 27]. There is an open question as to whether to achieve beneficial outcomes Wnt signalling needs to be therapeutically increased or decreased. Additionally, prediction tools required to assist personalised clinical Wnt treatment decisions are lacking [4, 27]. To address these contradictory reports we examined the role of both Wnt/β-catenin signalling activation and inhibition. We did so within the same cancer cell lines, avoiding any cell-type-specific or methodological differences which could be the reason behind the various contradictory studies. We focused primarily on the childhood cancer neuroblastoma, but also extended our findings to malignant melanoma and colorectal cancer.

A recent study of neuroblastoma survival rates in Europe between 1999 and 2007 showed no improvement [30], despite intensive research efforts. This lack of success is primarily attributable to the MYCN oncogene, which regulates numerous aspects of neuroblastoma development and progression [31]. MYCN is broadly considered un-druggable, and amplification of this oncogene is associated with drug resistant fatal high-risk neuroblastoma [31, 32]. We previously showed that pharmaceutical activation of Wnt/β-catenin signalling induced neuroblastoma cell death, but that differentiated neurons were spared [1]. Additionally, MYCN amplified (MNA) neuroblastoma cells were preferentially susceptible to Wnt activation [1]. While we showed that Wnt activation reduced the mRNA level of the MYCN oncogene, a direct functional link between MYCN and Wnt signalling has not yet been identified. Therefore, in addition to further investigating the role of Wnt activation and examining the effect of Wnt inhibition we profiled MYCN expressing neuroblastoma cells with a range of omic techniques and investigated the status of the Wnt signalling pathway.

Malignant melanoma is the most deadly form of skin cancer and its global incidence continues to increase [33, 34]. While recent therapeutic advances have led to improved survival, prognosis is still poor for most patients with metastases [35]. There exists an urgent requirement not only for novel single-agent therapeutics but also combination treatments, as tumours rapidly evolve drug resistance to existing therapies, e.g. BRAF (V600) inhibition and RAF/EGFR or RAF/MEK inhibitor co-treatments [36, 37]. Wnt/β-catenin signalling is associated with the melanocyte tissue of origin, the neural crest [38] and has been implicated in melanocyte development [39] and the oncogenesis and progression of malignant melanoma [2, 40–42]. The prominent role of β-catenin in malignant melanoma has been confirmed by genetic studies [2]. However, the utility of small molecule β-catenin inhibitors as therapeutic agents for this cancer or indeed the effect of pharmacological Wnt activation has not yet been investigated.

Colorectal cancer (CRC) has one of the highest incidences of all cancers [43, 44]. Despite the molecular pathogenesis and driving mutations of CRCs being well understood, CRC has a disease specific mortality rate of 33% in the developed world [43, 45, 46]. Therefore novel therapeutic approaches to CRC are urgently required. Wnt/β-catenin signalling is a key driver of CRCs [6, 16, 47–54], with activating mutations being present in over 70% of patients, and is therefore an attractive therapeutic target [16, 55]. While the importance of canonical Wnt/β-catenin signalling for CRC oncogenesis and progression has long been recognised [16, 50], and Wnt pathway activation is nearly a universal feature of all CRCs [55], there is currently no clinical use for APC or β-catenin mutations for early cancer detection or treatment selection as proven Wnt directed clinical therapies are lacking [4, 27, 55].

There is an unmet clinical need for novel therapies in each of these three cancer entities, with the targeting of the Wnt pathway representing a potentially useful strategy. However, a greater understanding of the effect of Wnt signalling modulation is required. In this study we revealed that both Wnt inhibition and hyper-activation are therapeutically valid approaches to neuroblastoma, once high levels of modulation are achieved, with each resulting in distinct beneficial outcomes. We also report numerous novel functional interactions between the MYCN and Wnt pathways. In line with the rapid advance of precision medicine in clinical oncology [56], to help potentially assist future treatment decisions we also used our functional transcriptomic readouts to inform the construction of a β-catenin gene signature predictive of neuroblastoma patient outcome. Finally, we extended our findings to show that Wnt/β-catenin signalling is not just a bi-directional vulnerability of neuroblastoma cells, but also of malignant melanoma and even β-catenin driven colorectal cancer cells.

## RESULTS

### Wnt/β-catenin signalling constitutes a therapeutically targetable vulnerability of MNA neuroblastoma

**Figure 1:**
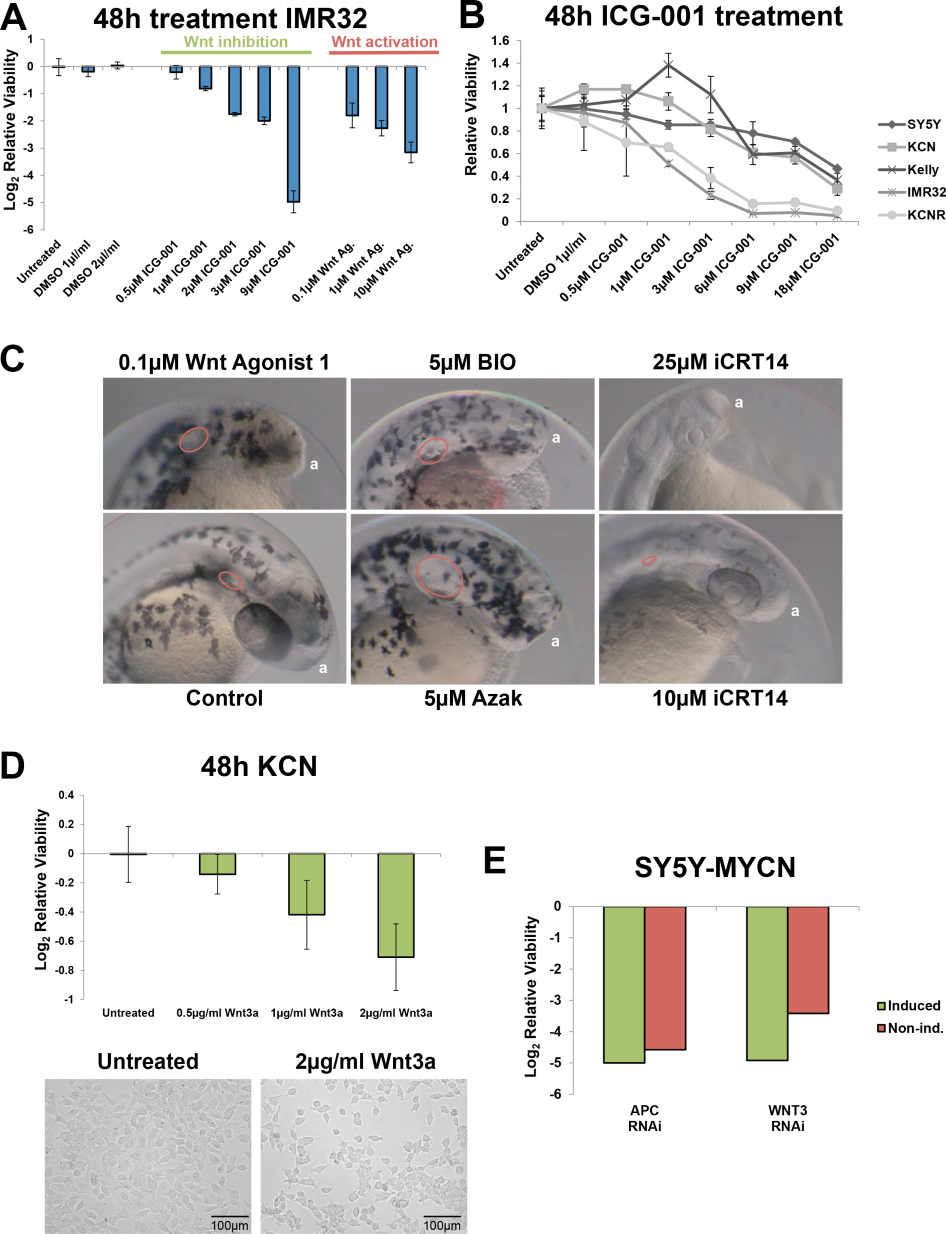
Wnt pathway modulation reduces neuroblastoma viability (**A**) Relative cell viability in IMR32 cells upon Wnt inhibition (ICG-001) and activation (Wnt agonist 1), after 48 h treatments as measured by MTS assay. (**B**) Relative viability of neuroblastoma cell line panel treated with the Wnt inhibitor ICG-001 for 48 h, as assed by MTS assay and is shown relative to viability of the corresponding control cells. (**C**) Zebrafish embryos treated for 48 h (4 hours post fertilisation [hpf]- 52hpf) with Wnt inhibitor (iCRT14) or activators (Wnt agonist 1, BIO and Azak). Anterior pole denoted by ‘a’, magnification is 10×, images from 52hpf. Melanocytes appear black due to their endogenous melanin. Otic vesicles, where visible, are circled in red. (**D**) Treatment with Wnt3a recombinant protein for 48 h reduces the viability of KCN cells. Top: MTS assay, bottom imagining. (**E**) RNAi screen viability results for APC and WNT3 knockdown in SY5Y-MYCN cells, with and without induction of the MYCN transgene. In induced cells MYCN overexpression was initiated 24 h prior to and maintained throughout the 72 h RNAi treatment. The screen and viability measurements are described in the Methods section.

To address the conflicting literature reports and assess whether both Wnt activation and inhibition can drive similar outcomes in the same neuroblastoma cell lines we treated IMR32 cells with the small molecules ICG-001 and Wnt agonist 1. ICG-001 inhibits β-catenin functioning by inhibiting β-catenin from binding to CBP (a rate-limiting factor in many transcriptional events), therefore acting as an inhibitor of the canonical Wnt signalling pathway [57]. Conversely, Wnt agonist 1 activates canonical Wnt signalling, inducing β-catenin/TCF-dependent transcriptional activity but not inhibiting GSK3β activity [58]. Both Wnt activation and inhibition strongly reduced IMR32 cell viability (Figure 1A), with inhibition producing the strongest reduction in cell viability by 48 h of treatment. We next confirmed that pharmacological Wnt inhibition reduced neuroblastoma cell viability in a dose-dependent manner across a panel of neuroblastoma cell lines (Figure 1B, Supplementary Figure S1A). The highest MYCN expressing lines IMR32 and KCNR were the most sensitive to ICG-001. Interestingly, these Wnt inhibition results mirror our previous findings for Wnt activation in this neuroblastoma cell line panel [1] with the addition of NBL-S and BE(2)-C cells (Supplementary Figure S1A), revealing that both pharmacological inhibition and activation of Wnt/β-catenin signalling impairs neuroblastoma cell viability.

Given the similar outcome of Wnt activation and inhibition on neuroblastoma cell viability we tested these small molecules in a zebrafish developmental screen to confirm that they can produce opposing effects. GSK3 inhibitors (BIO and azakenpaullone) were also used as a positive control for Wnt activation phenotypes, as GSK3 negatively regulates Wnt signalling by promoting β-catenin protein degradation [3], and as we have previously characterised the effect of these inhibitors on neuroblastoma cell viability [1]. Wnt activation with Wnt agonist 1 or GSK3 inhibitors in zebrafish embryos enlarged the otic (auditory) vesicles and expanded and disorganised the melanocyte (pigment cell) population (Figure 1C). Conversely, Wnt inhibition with iCRT14 reduced otic vesicle size and prevented melanocyte development (Figure 1C). The otic vesicle, melanocytes and neuroblastoma cells all originate from the neural crest. Wnt signalling is known to control numerous aspects of neural crest development [38, 59–62]. For the zebrafish assays we used the canonical Wnt inhibitor iCRT14 [63] as this molecule has a lower molecular weight than ICG-001 and had better penetrance of zebrafish chorions and embryos. Similar to ICG-001, iCRT14 reduced the viability of MYCN amplified neuroblastoma cells (Supplementary Figure S1B). Together, the zebrafish results confirmed that Wnt activation and inhibition with small molecules can drive opposing phenotypes.

Providing further evidence of the specificity of Wnt agonist 1, Wnt activation also disrupted anterior development, inducing a headless phenotype (Figure 1C). This is in line with Wnt's known role in anterior-posterior specification [58, 64, 65].

We next confirmed that modulation of Wnt signalling in either direction can reduce neuroblastoma cell viability using non-pharmaceutical approaches. When KCN cells were treated with Wnt3a protein they showed a dose dependent loss of cell viability (Figure 1D). The Wnt3a ligand was selected as we found that the activity of its target genes was modulated in response to MYCN status (see below). To further validate the role of β-catenin we transiently overexpressed a truncated mouse β-catenin/Tcf3 fusion gene, which is constituently active as it lacks regions associated with β-catenin degradation. The fusion gene also reduced MNA neuroblastoma cell viability with MYCN single copy SY5Y cells being more resistant (Supplementary Figure S1C), demonstrating a selective deleterious role of ectopic Wnt pathway hyper-activation in MNA neuroblastoma cells.

We next mined Wnt gene related data from our previously performed RNAi screen targeting the druggable genome [66] in the neuroblastoma cell line SY5Y-MYCN, in which MYCN overexpression can be induced [1, 67]. Knockdown of either WNT3, an activator of the canonical Wnt/β-catenin pathway, or APC, an inhibitor of the pathway, strongly (greater than 3 stand deviations from the median of the data distribution for the entire RNAi library) reduced cell viability (Supplementary Figure 1E), confirming the vulnerability of these cells to the modulation of the Wnt pathway in either direction. Interestingly, these genes had opposing correlations to patient survival (Supplementary Figure S1D, Supplementary Table S1), as determined using the R2 : Genomics Analysis and Visualization Platform (http://r2.amc.nl) in two large neuroblastoma tumour datasets (SEQC [68] and Kocak [69], 498 and 649 tumours respectively).

Furthermore, the knockdown of numerous Wnt associated genes altered the viability of SY5Y-MYCN cells (Supplementary Figure S2A). Interestingly, knockdown of some Wnt activating components enhanced viability (e.g. Wnt7A and FZD10), while others reduced it (e.g. WNT3 and FZD1), indicating that specific receptor-ligand combinations may drive opposing cell fates. Consistent with this result, different Wnt ligands/receptors were correlated with opposing prognosis in the R2 neuroblastoma patient datasets (Supplementary Table S1). These finding may reflect differing functions between canonical (β-catenin pathway) and non-canonical (planar cell polarity and calcium pathways) Wnt signalling. Wnt planar cell polarity has recently been associated with neuroblastoma cell viability and patient outcome [70].

A number of Wnt components also showed MYCN dependent effects in the RNAi screen (e.g. WNT3, FZD7 and LEF1), with responses to the knockdown of these genes varying between the un-induced and induced MYCN overexpression conditions. Further supporting its therapeutic potential, β-catenin was also an inferred transcriptional regulator (ITR, see below) of the 674 genes that strongly reduced SY5Y-MYCN cell viability when knocked down in the high-throughput RNAi screen (Supplementary Figure S2B). In summary, the pharmacological, recombinant protein, overexpression and RNAi screen results confirm that either Wnt activation or inhibition can decrease neuroblastoma cell viability.

### Differing dynamics of Wnt/β-catenin signalling activation and inhibition on neuroblastoma cell viability

**Figure 2:**
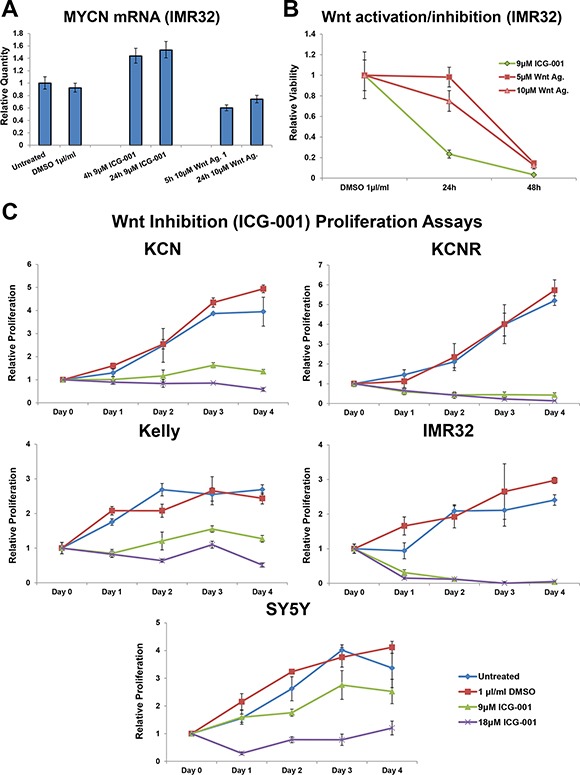
Varying dynamic response to small molecule Wnt activation/inhibition (**A**) Relative expression level of MYCN mRNA in IMR32 cells upon treatment with ICG-001 or Wnt agonist 1, as determined by qPCR. (**B**) Temporal profile of viability loss upon Wnt activation (Wnt agonist 1) and inhibition (ICG-001), as measured by MTS assay and relative to control cells. (**C**) Proliferation in response to four day Wnt inhibition (ICG-001) in MYCN amplified KCN, KCNR, Kelly and MYCN single copy SY5Y cells, as measured by MTS assay. Proliferation is relative to the corresponding Day 0 (pre-treatment) cells.

Having established that the observed reductions in viability were indeed Wnt specific, we next examined the cellular response to Wnt inhibition and activation in more detail. Wnt inhibition increased MYCN mRNA levels, whereas Wnt activation decreased them (Figure 2A). Neuroblastoma cell viability was reduced more quickly by Wnt inhibition than by Wnt activation (Figure 2B), suggesting differing mechanisms of reducing cell viability. Indeed, Wnt agonist 1 reduced viability primarily through apoptosis [1], while by contrast, ICG-001 inhibited the proliferation of all cell lines tested (Figure 2C). While Wnt inhibition reduced proliferation in all neuroblastoma cell lines it only reduced cells below pre-treatment numbers in the highest MYCN expressing cell lines, IMR32 and KCNR (Figure 2C). To assess the robustness of this observation we performed a Spearman's Rho (correlation) analysis, correlating MYCN mRNA expression level with the relative viability of each cell line at the 4 day ICG-001 time-point. This revealed that a cell line's MYCN levels were correlated to its responsiveness to Wnt inhibition (9 μM ICG-001: *R* = −0.9, *p-value* = 0.03739).

### Novel MYCN functional interactions with the Wnt/β-catenin signalling pathway

Having shown that both inhibition and activation of Wnt/β-catenin signalling preferentially reduced cell viability of high MYCN expressing neuroblastoma cells, we next explored how oncogenic MYCN and Wnt are functionally linked. To achieve this we mined our omic datasets, consisting of RNA-seq, 4sU-seq (labelled) ChIP-seq and interaction proteomics data which we had generated to globally profile overexpressed and amplified MYCN's signalling networks [66, 71]. 4sU-seq is a metabolic labelling method that allows the specific isolation of newly synthesized transcripts [71, 72], thereby enhancing the detection of differentially expressed genes, particularly for early time-points. The omic data was generated from a MYCN overexpression time-course, using the MYCN inducible cell line SY5Y-MYCN, and a panel of cell lines with varying MYCN amplification status. The cell lines express a range of different MYCN levels, with the overexpression in SY5Y-MYCN cells achieving MYCN levels similar to the KCN MYCN amplified cell line (Supplementary Figure S3A).

**Figure 3:**
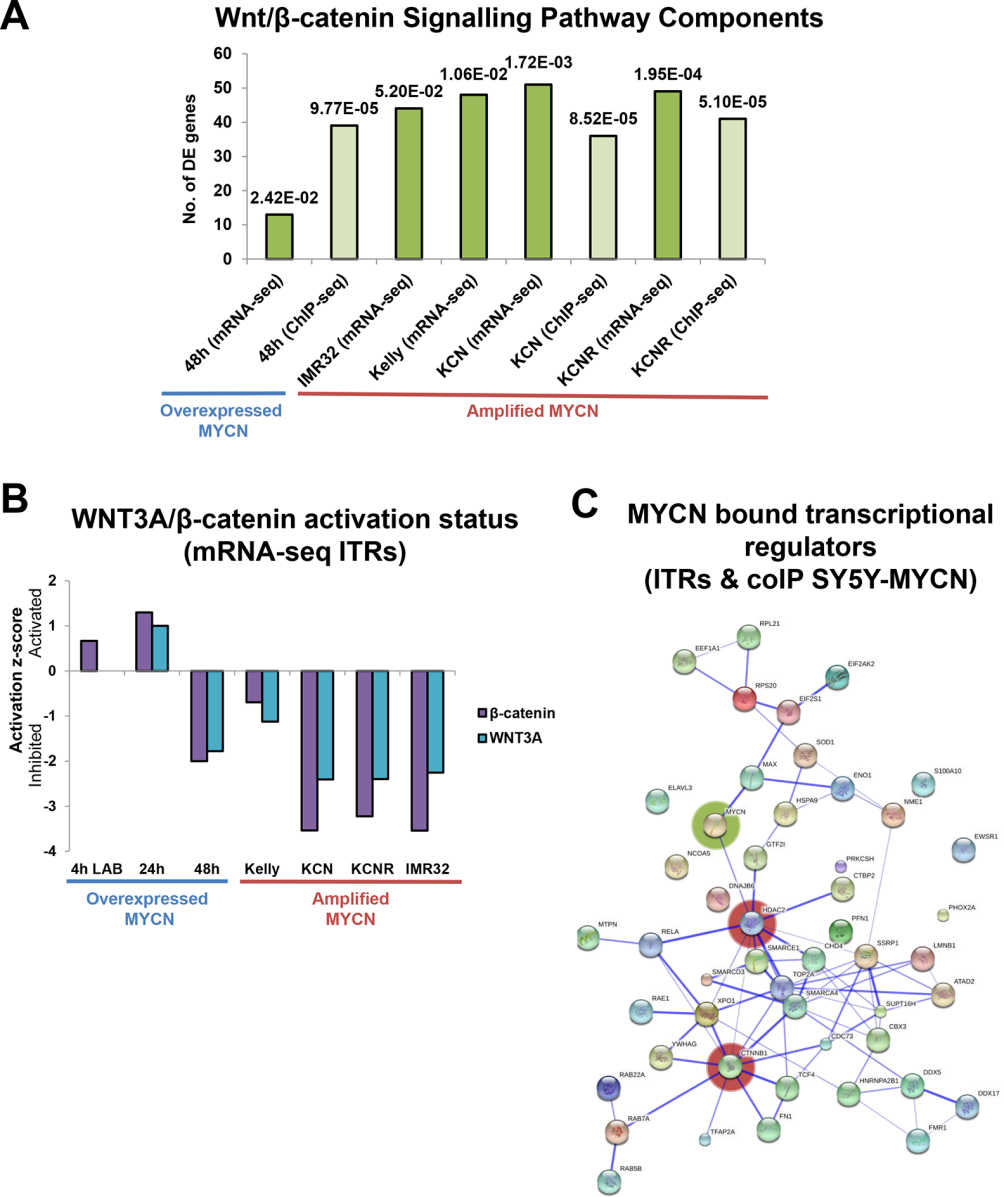
Omic scale investigation of MYCN interactions with the Wnt/β-catenin signalling pathway (**A**) Number of Wnt/β-catenin signalling component genes differentially expressed (mRNA-seq) or bound by MYCN protein (ChIP-seq), as identified by IPA. The pathway prediction *p-value* (overlap of known pathway genes and DE pathway genes) is indicated above each bar. Values are relative to those of the respective controls (MYCN overexpression time-points were compared with un-induced SY5Y-MYCN cells, while MNA lines were compared with single copy MYCN SY5Y cells). (**B**) Activation/inhibition z-score plot of WNT3A and β-catenin ITRs from mRNA-seq data. The 4 h LAB time-point is from 4sU-seq, whereas all other samples were generated using standard RNA-seq. (**C**) Protein interaction map (generated by String) of MYCN (coIP) bound proteins which were also ITRs (mRNA-seq), from SY5Y-MYCN cells. Proteins which were bound to MYCN at 4 h, 24 h or 48 h and were also an ITR at 4 h (labelled), 24 h or 48 h are included in the map. The most highly connected nodes are highlighted by red shading and MYCN by green.

We integrated the data from the disparate omic technologies using Ingenuity Pathway Analysis (IPA, http://www.ingenuity.com/), which allows the interrogation of high-throughput data at the pathway, network, function and regulator levels. Wnt/β-catenin signalling pathway components were significantly enriched in the differentially expressed genes of the MYCN overexpression time-course (24 h and 48 h) compared with un-induced cells (Figure 3A), as revealed by IPA. Wnt pathway components were also significantly enriched in the differentially expressed genes of each MNA cell line when compared with SY5Y, a MYCN single copy cell line (Figure 3A). This suggests extensive cross-regulation between these pathways with many Wnt pathway components being MYCN transcriptional targets. In order to identify direct MYCN targets we also performed MYCN ChIP-seq and found that the genes of numerous Wnt pathway members were directly bound by both the overexpressed and amplified MYCN oncogene (Figure 3A).

To assess the functional status of the Wnt pathway upon MYCN overexpression and amplification we used the differentially expressed gene lists to perform inferred transcriptional regulator (ITR) analysis, using IPA's ITRs algorithm [73]. Using prior knowledge this analysis identifies common transcriptional regulators known to be associated with the differentially expressed genes. ITR analysis revealed that both WNT3A and β-catenin were regulators of the genes differentially expressed in MNA cell lines compared with MYCN single copy cells, and in the MYCN overexpression time-course (Figure 3B, Supplementary Table S2A, S2B). A number of additional Wnt ligands (including WNT1 and WNT5A) were also regulators of the genes differentially expressed in these MNA cell lines (Supplementary Figure S3B). Supporting the ITR analysis we have shown that recombinant Wnt3a protein alters neuroblastoma cell viability (Figure 1D). However, in terms of autocrine signals WNT3 and WNT5A were the most highly expressed Wnt ligands across all five neuroblastoma cell lines.

Interestingly, ITR analysis showed that Wnt/β-catenin signalling was repressed in the MNA cells lines (Figure 3B, Supplementary Tables S2, S3). This repression is largely MYCN-dependent, as when MYCN overexpression was induced in the SY5Y-MYCN cells for 48 h WNT3A and β-catenin activity was inhibited. This inhibition occurred despite an initial activation of the WNT3A and β-catenin transcriptional regulators when MYCN overexpression was acutely induced, up to 24 h of overexpression. These results, based on the changes to the transcriptional status of all known Wnt target genes, suggest that sustained MYCN expression can repress Wnt/β-catenin functioning.

### MYCN and β-catenin signalling functionally cross-talk

In order to elucidate how MYCN represses Wnt/β-catenin target genes, we next examined the protein interactome of MYCN for Wnt related proteins. We determined the protein-protein interaction partners (interactome network) of overexpressed MYCN protein using mass spectrometry-based interaction proteomics, and assessed which putative interaction partners were also ITRs of the MYCN overexpression time-course RNA-seq. We thereby identified proteins which bound MYCN and transcriptionally regulated their target genes upon MYCN overexpression. A protein-protein interaction map of these regulators was then generated using the String database (www.string-db.org). This analysis revealed a small network, where β-catenin (CTNNB1) and HDAC2 were the best connected hubs (Figure 3C). HDAC2 provides a positive validation of this analysis approach as MYCN and HDAC2 have previously been shown to cooperate to repress gene expression [74]. However, until now protein-protein interactions between MYCN and HDAC2 was postulated by indirect evidence only [74]. The novel interaction between MYCN and β-catenin proteins, suggested by the interaction proteomics data, likely contributes to the observed changes in the transcriptional regulation of β-catenin target genes upon MYCN overexpression.

**Figure 4:**
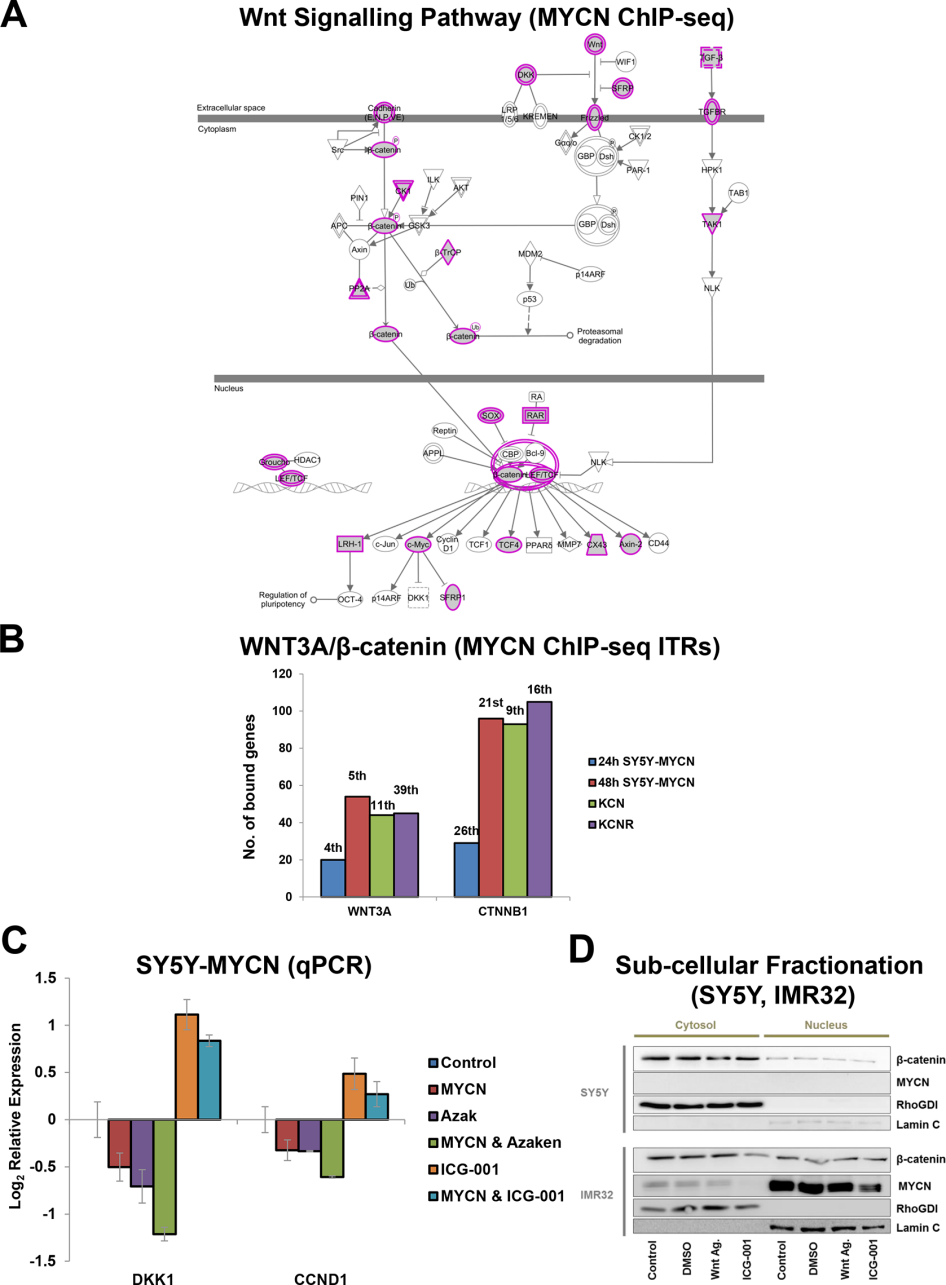
MYCN binds to genes of Wnt/β-catenin pathway components and their target genes (**A**) Wnt/β-catenin signalling pathway components genomically bound by MYCN as detected by MYCN ChIP-seq. Wnt/β-catenin pathway view with MYCN bound genes shaded grey with a pink outline. Single genes have a single pink line, while components representing gene families have a double pink line. (**B**) Number of genes bound by MYCN (ChIP-seq) contributing to WNT3A and β-catenin (CTNNB1) being predicted ITRs of the MYCNs genomic targets. Rank position (e.g. 1st) based on *p-value* of overlap, out of all ITRs identified from each time-point is shown above bars. (**C**) Effect on DKK1 and CCND1 mRNA levels of MYCN overexpression alone (48 h) or in combination with Wnt inhibition (48 h 9 μM ICG-001) or Wnt activation (24 h 1 μM Azaken), in low and high MYCN settings. (**D**) Sub-cellular fractionation showing nuclear and cytosolic localisation patterns of β-catenin and MYCN proteins in SY5Y and IMR32 cells. Cystosolic control RhoGDI and nuclear control Lamin C proteins are also shown. Experimental conditions were untreated control cells, or 3 h 1 μl/ml DMSO vehicle control, 1 μM Wnt Agonist 1 or 18 μM ICG-001 treated cells.

Together, these results suggested two mechanisms by which MYCN could inhibit Wnt/β-catenin signalling. Increased MYCN levels could bind and sequester β-catenin, thereby preventing it from activating target genes. Alternatively, β-catenin could direct MYCN to β-catenin target genes, resulting in altered regulation of these genes. The latter appears to be the case as MYCN (ChIP-seq) bound to a number of genes encoding Wnt/β-catenin pathway components (Figures 3A, 4A, Supplementary Table S4). Importantly, there was also an overlap between MYCN target genes (overexpressed and amplified MYCN target genes, bound by MYCN, as revealed by ChIP-seq) and known WNT3A and β-catenin target genes (Figure 4B, Supplementary Table S5). This, combined with the repression of known WNT3A/β-catenin target genes revealed by the transcriptomic analysis, suggests that β-catenin can direct MYCN to its genomic targets and supress normal β-catenin regulation of these genes. Supporting this, MYCN mediated transcriptional repression of certain targets was further enhanced by pharmacological activation of β-catenin function, while β-catenin inhibition reversed the direction of regulation to activation (Figures 4C, Supplementary S3C). In addition, both the WNT3A and β-catenin gene regions themselves were also bound by MYCN (Figure 4A, Supplementary Table S4), further supporting the hypothesis of numerous points of functional crosstalk between the MYCN and Wnt pathways via β-catenin.

Cell fractionation analysis revealed that both MYCN and β-catenin are present in the nuclei of IMR32 cells, which further supports our hypothesis that β-catenin protein and elevated MYCN protein interact in the nucleus. While nuclear β-catenin is generally considered a readout for Wnt signalling activation, our findings suggest that MYCN-β-catenin protein interaction reverses the normal transcriptional regulation of β-catenin target genes. Neither ICG-001 nor Wnt Agonist 1 treatment strongly altered nuclear β-catenin levels, which is in line with the drugs' mode of action: blocking β-catenin from binding to its nuclear co-factors rather than regulating β-catenin sub-cellular localisation. However, interestingly, ICG-001 treatment did reduce both cytosolic and nuclear MYCN levels (Figure 4D). This ICG-001-mediated MYCN regulation occurs through protein level mechanisms, as MYCN mRNA levels were not reduced by ICG-001 treatment, thereby revealing yet another level of β-catenin-MYCN protein cross-talk. Finally, as ICG-001 actually increased MYCN mRNA levels (Figures 2A and Supplementary S3C) while reducing MYCN protein levels, these results further support the existence of a converse regulatory buffering mechanism between MYCN mRNA and protein levels which we previously identified [1].

### Neuroblastoma cell line omic findings are applicable to clinical patient samples

The above data were obtained with neuroblastoma cell lines and hence may be cell line specific or may represent adaptations to cell culture conditions. Therefore, we examined whether these results were more generally applicable to neuroblastoma tumour biology by testing the salient findings in a well-characterised neuroblastoma tumour microarray dataset comprising 478 patients [75].

**Figure 5:**
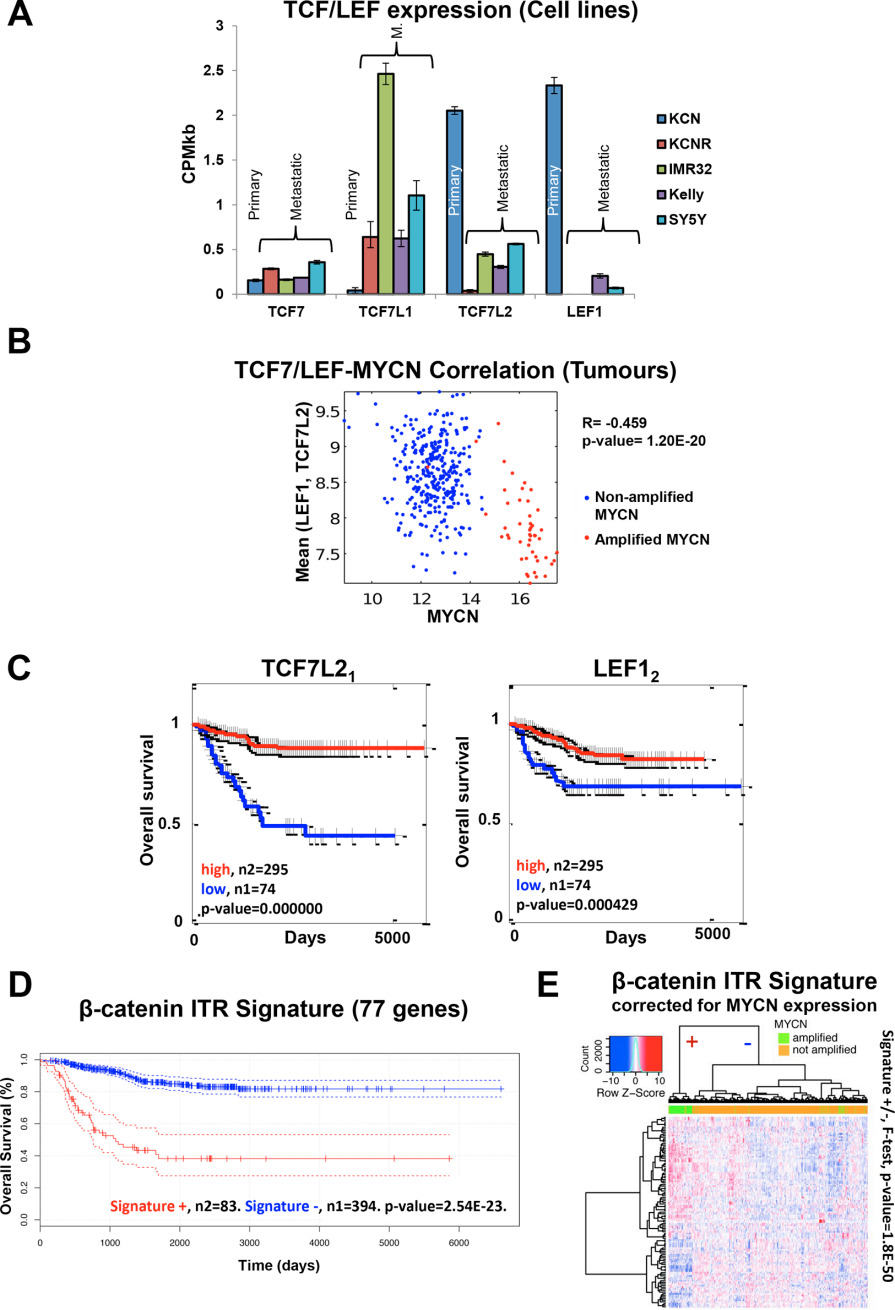
Cell line omics data is predictive of neuroblastoma patient expression profiles and outcome (**A**) TCF/LEF mRNA expression across the cell lines (mRNA-seq). (**B**) Negative correlation between MYCN mRNA and mean LEF1, TC7L2 mRNA levels in tumour samples. R denotes the Pearson product moment correlation coefficient. (**C**) TCF7L2 and LEF1 mRNA expression is predictive of neuroblastoma patient outcome. Subscript after gene name denotes the specific microarray probe. (**D**) The DE genes contributing to the identification of β-catenin as an ITR of the mRNA-seq data were used to generate a gene signature. The β-catenin activity signature was predictive of neuroblastoma patient outcome. (**E**) Heat map of the β-catenin ITR signature for the MYCN expression corrected mRNA levels from the patient microarray data. The dendrogram on the top shows the separation of Signature (+) and Signature (−) for all tumours, a clear separation between MYCN amplified and not amplified tumours can be seen (*F*-test, *p-value* = 1.8e-50). All 117 probe sets for the 77 genes are significantly differentially expressed (*p*-adj < 0.05) between MYCN amplified and MYCN non-amplified tumours.

As determined by ChIP-seq, MYCN bound to three of the four TCF/LEF genes (Figure 4A, Supplementary Table S4), which are the transcription factor effectors of Wnt signalling. The TCF/LEF genes bind nuclear β-catenin as a transcriptional co-factor. The expression of these genes was also differentially regulated across the cell lines (Figure 5A). TCF7L1 expression was upregulated in lines derived from metastatic tumours compared to primary tumours, while conversely both TCF7L2 and LEF1 were strongly downregulated in the metastatic lines (Figure 5A). LEF1 was silenced in KCNR and IMR32 cells (0 CPMkb) and was the 4th most highly downregulated gene (15.38 log_2_FC) between the patient-matched lines, KCN and KCNR. In addition, LEF1 was identified as a top transcription factor associated with overexpressed MYCN's genomic targets using the DiRE [76] tool (Supplementary Figure S4A). Therefore, MYCN and LEF1 share a number of transcriptional targets, possibly acting as co-regulators.

The negative correlation between MYCN expression and LEF1 and TCF7L2 expression seen in the cell lines was also observed in the neuroblastoma tumours (Figure 5B), confirming the functional MYCN-Wnt interactions revealed in the cell line data. Therefore, we tested whether TCF/LEF mRNA expression can segregate the survival of the neuroblastoma patient cohort (Figure 5C). Consistent with the cell line data, TCF7L2 and LEF1 could predict patient survival, with low expression indicating worse outcome. By contrast, TCF7L1 expression was not predictive of survival (Supplementary Figure S4B), suggesting that the different TCF/LEF genes have heterogeneous functions. These data support the finding of the omics and RNAi screen that Wnt signalling plays highly specific roles in neuroblastoma biology. Further investigation of specific Wnt ligand/receptor/transcription factor combinations is required to fully unravel this complexity.

Given this complexity, we wanted to corroborate the value of TCF7L2 and LEF1 expression for patient stratification. If they are true predictors of patient survival, the genes that they regulate should also be predictive. Therefore, we generated a gene signature using the differentially expressed genes that are regulated by their co-factor β-catenin according to the ITR analysis of the cell lines (Supplementary Table S6). Only genes which were differentially expressed in at least two cell lines were used. This signature showed predictive ability, independently segregating patient survival cohorts (Figure 5D). This signature could discriminate between MYCN-amplified and non-amplified tumours even when corrected for MYCN effects (*F*-test, *p-value* = 1.8E-50. Figures 5E, Supplementary S4C). Furthermore, while the β-catenin ITR signature identified poor prognosis MYCN amplified patients it also identified an additional poor survival cohort not harbouring elevated MYCN expression (Supplementary Figure S4D). While the β-catenin ITR signature captures MYCN-amplified patients it also contains additional survival prediction capability beyond that of MYCN expression alone (Supplementary Figure S4D, S4E). Thus, our systems level analysis identified a specific β-catenin signature that can correctly stratify patients in terms of prognosis, and can potentially also be used to stratify patients into treatment groups once Wnt modulation therapies enter clinical use.

### Wnt signalling contributes to neuroblastoma stemness/differentiation status

Having identified cross-talks between MYCN and Wnt/β-catenin signalling and the relevance of Wnt/β-catenin signalling to patient outcome, we next examined the cellular phenotypes induced by Wnt inhibition (ICG-001) and activation (Wnt agonist 1 treatment) in more detail. IMR32 cells were treated with concentrations of each drug that had resulted in a similar reduction of viability by 48 h treatment (Figure 1A), and the resulting changes in cell shape were imaged (Supplementary Figure S5A). Wnt activation resulted in cells retracting their neurites and becoming rounded, a process not mirrored by Wnt inhibition. Although Wnt activation was slower at reducing cell viability than Wnt inhibition (Figure 2B), the phenotypic changes to cell shape occurred more rapidly, being visible within 18 h of Wnt agonist 1 treatment (Supplementary Figure S5B).

The imaging suggested that Wnt activation was directing the cells to become more blast-like, rounded cells with a retraction of cellular protrusions, while Wnt inhibition seemed to be promoting the cells towards differentiation, blocking proliferation and increasing axon outgrowth. Therefore, we next evaluated the effect of co-treatment of these inhibitors with Retinoic Acid (RA), which is used clinically to differentiate neuroblastoma cells into benign terminally differentiated neurons [77–80]. While RA treatment is successful for a majority of low-risk neuroblastoma patients, MNA tumours are generally resistant to its effects. We therefore performed the combination treatments in the MNA IMR32 cell line.

**Figure 6:**
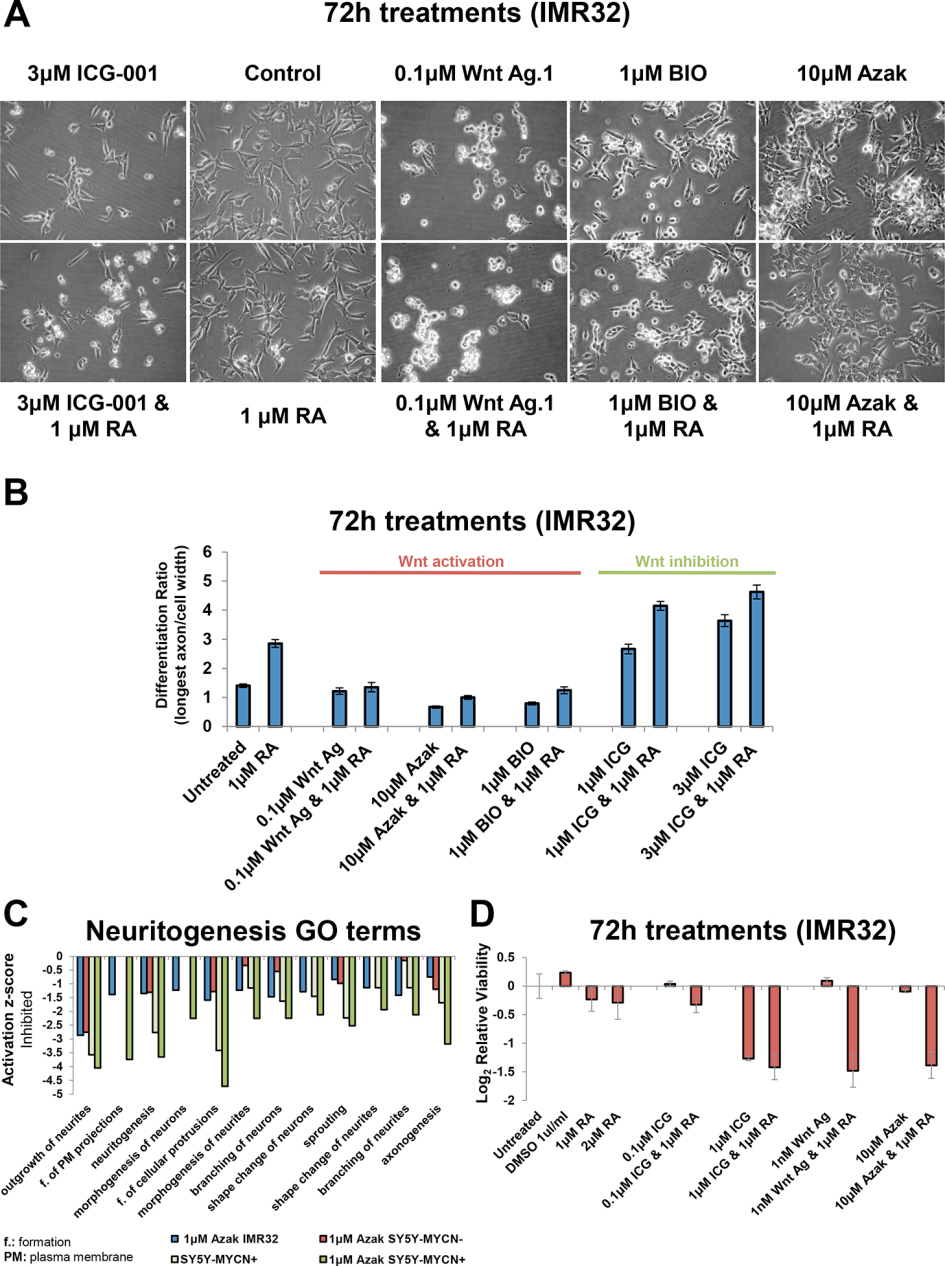
Wnt inhibition promotes differentiation while activation promotes stemness (**A**) Imaging of IMR32 cells treated for 72 h with Wnt inhibitor (ICG-001) or Wnt activators (Wnt agonist 1, azakenpaullone and BIO) singly, or in combination with Retinoic Acid (RA). All panels are imaged at 40x magnification. (**B**) The differentiation ratio of IMR32 cells treated for 72 h with individual agents or combination treatments with RA, was calculated by dividing the length of the longest axon of a cell by the cell's width. Measurements made using ImageJ v1.44p (http://imagej.nih.gov/ij). Range of measured cells (N) per treatment group is 50-171. Error bars depict the standard error of the mean. (**C**) IPA disease and function GO term analysis of RNA-seq samples revealed that differentially expressed genes upon 24 h azakenpaullone treatment (in IMR32 or SY5Y-MYCN) or 48 h MYCN overexpression (SY5Y-MYCN), and combination treatment (SY5Y-MYCN) were enriched for differentially expressed genes associated with the inhibition of neuritogenesis. SY5Y-MYCN- denotes un-induced cells, whereas SY5Y-MYCN+ refers to cells in which MYCN overexpression was induced for 48 h. Values are relative to those of the respective controls (IMR32 and SY5Y-MYCN untreated cells). (**D**) Cell viability analysis of IMR32 cells treated for 72 h with individual agents or combination treatments with RA, as detected by MTS assay.

Cells not killed by Wnt inhibition (ICG-001) were more differentiated (Figure 6A, 6B), as measured by the ratio of the longest axon divided by the cell width. Indeed, ICG-001 only treatment had a stronger effect than RA only treatment. ICG-001 treated cells extended their axonal protrusions and became more elongated (Supplementary Figure S6A). Upon co-treatment with RA, Wnt inhibited cells became significantly more differentiated than with ICG-001 treatment alone (*t-test*: 3 μM ICG-001, *p* = 0.0019; 1 μM ICG-001, *p* = 0.0001) (Figure 6A, 6B and Supplementary S6A). This suggests that Wnt inhibition and RA cooperate to overcome the resistance of MNA cells to differentiation therapy.

Conversely, Wnt activated cells (Wnt agonist 1) became more stem-like with a significant reduction of axon length and cell width (*p* = 0.0002 and 0.0005 respectively) compared with untreated cells (Figure 6A, 6B and Supplementary S6A). Wnt activation using the GSK3 inhibitors azakenpaullone and BIO also induced the cells to adopt a more stem-like morphology (Figure 6A, 6B), strongly reducing axon length (Supplementary Figure S6A). The effects of Wnt agonist 1 were not rescued by RA co-treatment (axon length *p* = 0.2024; cell width *p* = 0.1977). However, RA co-treatment could slightly, but significantly, mitigate the effect of azakenpaullone and BIO (*p* = 0.0001 and 0.0004, respectively) but not return axon length to the level seen in control cells, yet alone that of RA only treated cells.

We next performed transcriptomic (RNA-seq) data of cells treated with 1 μM azakenpaullone for 24 h to examine the underlying molecular changes driving the Wnt activation stem-like phenotype. Azakenpaullone was selected as this drug could induce morphological changes without causing a reduction in cell viability. RNA-seq was performed in SY5Y-MYCN cells (un-induced and 48 h MYCN overexpressing), and combined with previously generated RNA-seq data from IMR32 cells [1]. IPA analysis of genes differentially expressed upon azakenpaullone treatment in IMR32 cells and SY5Y-MYCN cells, compared to their respective controls, revealed an enrichment of neuritogenesis related Gene Ontology terms and showed that neuritogenesis was inhibited (Figure 6C, Supplementary Table S7). All terms showed inhibition of neuritogenesis with azakenpaullone or MYCN overexpression treatment, compared to their untreated counterparts. Combination treatment produced the greatest reduction (Figure 6C), suggesting that MYCN and activated Wnt (azakenpaullone) can work together to induce a more stem-like state. In addition, genes differentially expressed by azakenpaullone treatment were associated with the inhibition of differentiation associated processes (Supplementary Figure S6B).

### Wnt modulation and retinoic acid act synergistically to reduce cell viability

We next assessed the effect of Wnt and RA co-treatments on cell viability. RA co-treatment had little effect on the higher ICG-001 and Wnt agonist 1 concentrations which strongly reduced cell viability (Supplementary Figure S6C). However, RA co-treatment sensitised IMR32 cells to low dose treatments (Figure 6D). Even 1 nM of Wnt agonist 1, which on its own had no effect on viability, strongly synergised with RA co-treatment reducing viability by 64% (Figure 6D). Similarly RA and 10 μM azakenpaullone were synergistic, reducing cell viability by 61%, despite treatment with azakenpaullone alone reducing cell viability by a mere 6% (Figure 6D). Collectively, these results suggest that RA-Wnt combination treatments could be a far more effective therapy for MNA neuroblastoma than the current clinical treatment regime with RA only. In addition, Wnt agonist 1 is a promising lead compound, showing activity in the nanomolar range, with even 1 nM strongly affecting MNA cells when combined with RA.

We next examined whether cells recovered rapidly from combination treatment, or if there was a longer term effect once the small molecules were removed. As before, IMR32 cells were treated for 72 h. However, the treatment was then ended and the cells were grown in normal media for a further 72 h before cell viability was measured. For both Wnt inhibition and Wnt activation RA co-treatments cells did not recover, despite the removal of the small molecules (Supplementary Figure S6D). In fact, a greater loss of viability was observed than when viability was assessed immediately at the end of the 72 h drug treatment (Supplementary Figure S6C, S6D), dramatically so in the case of the Wnt agonist 1 and RA co-treatment. Over this time period RA sensitised the cells to ICG-001 and Wnt agonist 1 treatments, further reducing the viability compared with single agent treatments (Supplementary Figure S6D). Such RA-mediated sensitisation to Wnt treatment was not observed in MYCN single-copy SY5Y cells (Supplementary Figure S6E). These results provide further evidence that combination Wnt-RA treatments hold great potential for the treatment of MNA neuroblastoma.

### Wnt signalling is a bi-directional vulnerability of additional cancer entities

**Figure 7:**
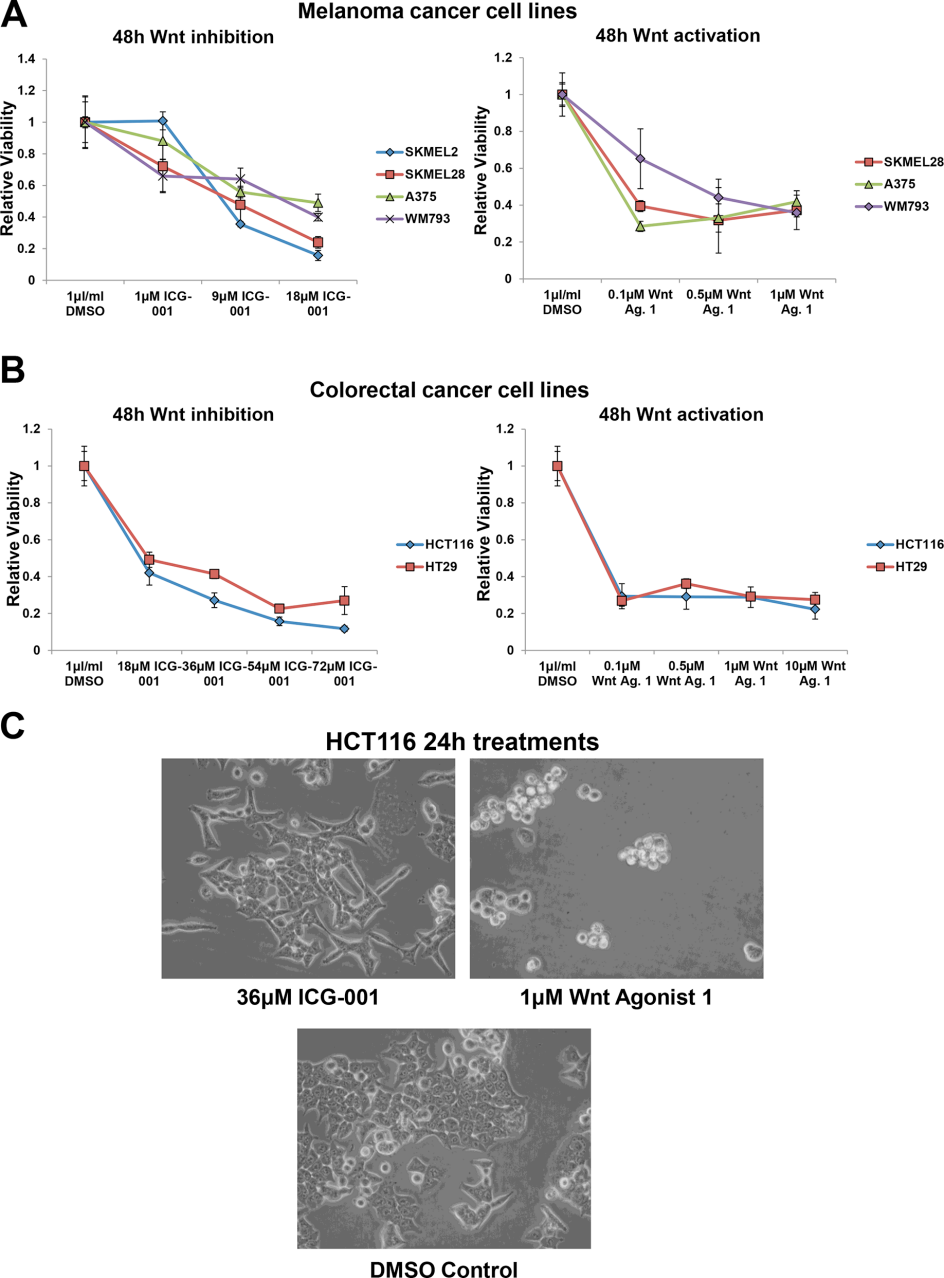
Wnt signalling is also a bi-directional vulnerability in malignant melanoma and colorectal cancer cells (**A**) Cell viability analysis of the dose response of a panel of malignant melanoma cell lines treated for 48 h with ICG-001 or Wnt agonist 1, as detected by MTS assay. (**B**) Cell viability analysis of the dose response of colorectal cancer cell lines (HCT116 and HT29) treated for 48 h with ICG-001 or Wnt agonist 1, as detected by MTS assay. (**C**) Imaging of HCT116 cells treated for 24 h with Wnt inhibitor, ICG-001 or Wnt activator, Wnt agonist 1. All panels are imaged at 40x magnification.

We next determined whether the Wnt signalling bi-directional vulnerability is unique to neuroblastoma or if it is applicable to other cancers. To test this hypothesis we selected two cancer types, malignant melanoma [2, 15, 40–42] and CRC [6, 16, 47–54], where constitutive activation of Wnt/β-catenin is a key oncogenic driver. Hence one would expect Wnt inhibition but not hyper-activation to have therapeutic potential. However, both inhibition (ICG-001) or hyper-activation (Wnt agonist 1 and BIO) of Wnt signalling reduced cell viability by a comparable degree in all melanoma and CRC cell lines tested (Figure 7A, 7B and Supplementary Figure S7A, S7B). HCT116 has an activating mutation in β-catenin, while in HT29 β-catenin is activated through an inactivating mutation in APC (COSMIC database v73 http://cancer.sanger.ac.uk/cosmic). Therefore, despite both CRC cell lines featuring activation of β-catenin signalling by different mechanisms, Wnt hyper-activation (Wnt agonist 1 and BIO) or inhibition (ICG-001) strongly reduced cell viability, suggesting that β-catenin signalling needs to be finely balanced for the cell to derive a survival and proliferation advantage. This may be a reason for a large spectrum of mutations that activate β-catenin signalling to different strengths [81].

Akin to the situation in neuroblastoma, the role of Wnt in melanoma is complex. Activated Wnt signalling drives melanoma formation, but is associated with better prognosis [2, 10, 82]. β-catenin also regulates the metastatic potential (in a PTEN loss/BRAF activating mutation context-dependent manner) and differentiation of melanomas [2]. In the presence of PTEN/BRAF mutations activated β-catenin drives highly metastatic tumours. Yet even SK-MEL-28, a cell line harbouring both PTEN (missense) and BRAF (V600E activating) mutations (COSMIC database v73 http://cancer.sanger.ac.uk/cosmic), was susceptible to Wnt hyper-activation, as well as inhibition (Figure 7A and Supplementary S7A). Indeed, prolonged Wnt activating or inhibiting treatments resulted in the same degree of viability loss, with the near total abolition of SK-MEL-28 cells (Supplementary Figure S7C). Both HCT116 and A375 cells had high levels of nuclear β-catenin and levels of nuclear β-catenin were not strongly altered upon ICG-001or Wnt Agonist 1 treatment (Supplementary Figure S7D), in line with the mode of action of these drugs: blocking β-catenin from binding to its nuclear co-factors rather than regulating β-catenin sub-cellular localisation.

Imaging of treated CRC cells revealed that, as in neuroblastoma, Wnt agonist 1 treatment induced cell rounding and apoptosis, while ICG-001 primarily reduced cell viability by blocking proliferation (Figure 7C). Again, matched concentrations of Wnt agonist 1 and ICG-001 were used, which showed a comparable level of viability loss by 48 h (Figure 7B). Together, these data confirm that Wnt/β-catenin signalling is a bi-directional vulnerability of a number of discrete cancer entities, and not just a neuroblastoma specific phenomenon.

## DISCUSSION

### Neuroblastoma, melanoma and CRC are dependent on a baseline level of Wnt signalling and susceptible to its modulation

Wnts are one of the earliest signals activated during development [64, 83]. They therefore exert substantial control over cell fate decisions. Given the prominent role of Wnt signalling in cancer stem cells the pathway represents an attractive therapeutic target, especially if safety concerns can be allayed [26]. Wnts have well known regulatory roles in the neuronal lineage including in the neural crest, which is the neuroblastoma and melanocyte tissue of origin, and in controlling stem cell proliferation in colonic crypts [38, 84, 85]. While MYCs are known to be downstream targets of Wnt signalling, the level of crosstalk between MYCN and upstream Wnt signalling components [1, 86, 87] we observed was not expected. Importantly, Wnt signalling was independently highlighted by each of our omic experiments as a MYCN interacting pathway, providing strong evidence for close, multi-level interactions between MYCN and the canonical Wnt signalling pathway.

Our integrated MYCN transcriptomics and interactomics analysis highlighted β-catenin as a key hub gene in the MYCN interaction network. Deregulated β-catenin was previously associated with high-risk neuroblastoma without MNA [88]. Here, we show that β-catenin also has important functional relevance for MNA cells, representing a promising therapeutic target. We also revealed that MYCN binds to DNA in the region of the β-catenin gene, as well as numerous other Wnt pathway components, and that β-catenin inhibition reduces nuclear and cytosolic MYCN protein levels suggesting a circuitry of mutual regulation. These multiple Wnt-MYCN links were connected to patient outcomes, with TCF7L2 and LEF1 expression level, or β-catenin activity status being prognostic. TCF7L2 and LEF1 are likely involved in the genesis of neuroblastoma, similar to TCF7L2 driving c-MYC-dependent intestinal tumorigenesis [89], but subsequently were sharply downregulated in MNA tumours and cell lines from metastatic neuroblastomas. The switch we observed in TCF/LEF gene expression in neuroblastoma is likely to have functional consequences as different TCFs can act differentially on the same DNA binding site [90].

Wnt inhibitors inhibit colorectal cancer growth both *in vitro* and *in vivo* [57, 63], and are showing promise in other cancers including relapsed acute lymphoblastic leukaemia [91]. Conversely, Wnt activation is emerging as a potential treatment option for some cancers, including embryonal tumours [1, 11]. This dichotomy is resolved by the increasing evidence that a gene can behave as an oncogene in one tumour type (e.g. β-catenin in colorectal cancer, melanoma and good prognosis medulloblastoma), but act as a tumour suppressor in another (e.g. β-catenin in rhabdomyosarcoma) [2, 3, 11–13, 17]. However, even within the same cancer entity such as melanoma, both Wnt inhibition and activation have been reported to reduce tumour burdens *in vivo* in mouse models [2, 10]. Indeed, β-catenin also has contradictory roles in matched cancerous and normal tissues, where it maintains hyperproliferation or antagonises proliferation, respectively [14]. Our data expand this dual role, showing that Wnt activation or inhibition produces similar losses in cell viability even within the same cell type, albeit by different mechanisms and cell fates. These results also reconcile previously contradictory reports that Wnt inhibition [23, 24] or activation [1, 25] reduced neuroblastoma viability and raise the possibility that Wnt/β-catenin signalling represents a more general vulnerability of a large range of cancer types.

It has been suggested previously that for optimum therapeutic effect a fine balance between the cell proliferation and differentiation arms of the Wnt signalling pathway must be maintained [27]. However, our results offer an alternative view suggesting that rather than subtle adjustments, large disruptions to a cancer cell's balanced Wnt signalling will achieve optimum therapeutic effect by driving the cell to either undergo proliferation block and terminal differentiation (Wnt inhibition) or apoptosis (Wnt hyper-activation). Neuroblastoma, CRC and melanoma cancer lines seem to be tolerant of a defined range of Wnt signalling, with perturbation beyond this range in either direction being detrimental to cancer cell viability. While the phenomenon of oncogenic addiction is well documented [92, 93] the possibility of an upper threshold of activation for addicted cells has not previously been explored. We show that exceeding this threshold, at least for β-catenin, is detrimental to the cancer cell, representing an exploitable vulnerability.

Our finding that, despite their heterogeneous genetic backgrounds, neuroblastoma, melanoma and CRC cells are susceptible to either stimulation or inhibition of Wnt signalling could open several therapeutic options for these cancers. Our results suggest that both Wnt inhibition and activation therapies should be assessed in all of the many cancer types in which Wnt is involved [3, 4], as, counterintuitively, even CRC cells that are dependent on constitutively activated β-catenin signalling were susceptible to inhibition and hyper-activation of this pathway.

Our findings provide two opposing options for therapeutically targeting the Wnt/β-catenin signalling vulnerability in neuroblastoma, either of which will likely reduce MNA neuroblastoma tumour burdens. We also showed that combination treatment of RA and Wnt modulation acted synergistically. The combination of Wnt inhibition (ICG-001) and RA strongly reduced cell viability and pushed any surviving cells towards differentiation, suggesting that this treatment strategy could be of particular benefit therapeutically. RA also further sensitised MNA cells to Wnt activation (Wnt agonist 1). By combining Wnt agonist 1 with a proven neuroblastoma therapy [77–80] this co-treatment offers a wide therapeutic window and great promise for tackling MNA tumours. There is likely to be broader applicability of these findings as RA is a therapeutic agent used in the clinical treatment of a wide range of cancers, and has been investigated as a treatment for melanoma (with both completed and ongoing clinical trials, www.clinicaltrials.gov) and for CRC (pre-clinical models) [94–98]. RA and Wnt co-treatments may therefore prove equally synergistic for melanoma and CRC.

## MATERIALS AND METHODS

### Cell culture and treatments

For cell culture conditions and cell sources see Duffy et al. [1]. Additionally NBL-S (MYCN single copy, with somewhat elevated MYCN expression [99]) and BE(2)-C (MYCN-amplified) were used for IC-001 and Wnt Agonist 1 treatment. MYCN overexpression in the SH-SY5Y/6TR(EU)/pTrex-Dest-30/MYCN (SY5Y-MYCN) [1, 67] line was induced by 1 μg/ml Doxycycline (Sigma-Aldrich). SY5Y-MYCN media was supplemented with 7.5 μg/ml Blasticidine (Sigma- Aldrich) and 200 μg/ml G418 (Sigma-Aldrich). Small molecules used were: 1-azakenpaullone (Azak, Sigma-Aldrich), BIO-acetoxime (BIO, Tocris), Wnt agonist 1 (Merck), ICG-001 (Selleck), iCRT14 (Tocris), and Retinoic Acid (RA, Sigma-Aldrich). Stock solutions were dissolved in DMSO. Compounds were replenished every 24 h for any treatment longer than a 24 h duration.

KCN cells were treated with media containing human recombinant Wnt3a (R&D Systems). The Wnt3a- containing media was replenished 4 times over the course of the 48 h treatment. mβ-catenin/Tcf3 fusion construct (Genbank accession number KM189193) was transfected into the cells using jetPRIME (Polyplus), according to the manufacturer's instructions. Empty or fusion vector was transfected at 100 ng (KCNR) or 200 ng (Kelly) per well of a 96-well plate.

### RT-qPCR

RT-qPCR were performed as previously described [1]. Error bars on qPCR graphs depict RQ Min and RQ Max values. For TaqMan assays and primer sequences, used with TaqMan and SYBR reagents respectively (Applied Biosystems), see Supplementary Table S8.

### Cell fractionation and subsequent western blot analysis

Cells grown in 6-well plates were dissociated and then pelleted by 4 minutes of centrifugation at 2,000 rpm at +4°C. Upon removal of the media, cells were resuspended gently and lysed for 10 min on ice using 200 μl ice-cold Lysis buffer (40 mM Hepes, pH 7.5, 0.1% NP-40, 5 mM EGTA, 5 mM MgCl2, 1 MM DTT, phosphatase and protease inhibitors cocktail). Lysed cells were centrifuged for 10 minutes at 10,000 rpm at +4°C. This collected supernatant contains the cytosolic fraction. The pellet was washed once with 200 μl of the Lysis buffer and then resuspended in 100 μl of the ice-cold Nuclear extraction buffer (50 mm β-glycerophosphate, pH 7.3, 420 mM NaCl, 1.5 mM MgCl2, 0.2 mM EDTA, 1 mM EDTA, 1 mM DTT, 25% Glycerol). This fraction was sonicated by two 2 second pulses at 10% intensity with 2 second pause. Nuclear fraction was cleared from residual membranes by centrifugation at 14,000 rpm for 4 minutes. This collected supernatant contains the nuclear fraction.

Western blot analyses were performed as described by Duffy et al. [1]. All primary antibodies were incubated overnight at +4°C using a 1:1,000 dilution. Primary antibodies used are against MYCN (B8.4.B) (mouse, sc-53993, Santa Cruz), β-catenin (rabbit, 9562S, Cell Signaling Technologies), Rho GDI (A-20) (rabbit, sc-360, Santa Cruz), Lamin A/C (rabbit, #2032, Cell Signaling Technologies).

### Deep sequencing and bioinformatics analysis (mRNA-, miRNA- and ChIP-seq) and patient tumour microarrays

RNA-*seq* was conducted and subsequent bioinformatics analysis performed as previously described [1, 66, 71], for 5 neuroblastoma cell lines and the MYCN over expression time-course (un-induced, 1 h, 4 h and 24 h). In addition to standard mRNA-seq, 4sU labelling mRNA-seq was performed on some of the time-course samples (un-induced, 1 h and 4 h). These samples were incubated in 500 μM 4-thiouridine (4sU, Sigma) 30 minutes before cell lysis, to label only the mRNAs synthesised during that time. Each sample was separated into 3 pools, total, labelled and un-labelled (pre-existing) as previously described [100, 101]. In addition, untreated and 24 h 1 μM azakenpaullone treated IMR32 cells sequenced previously [1] were re-analysed for neuritogenesis and differentiation associated GO terms. Additional samples for 4 conditions (un-induced, 48 h overexpression, un-induced 24 h 1 μM azakenpaullone, and co-treatment: 24 h 1 μM azakenpaullone with 48 h overexpression) were sequenced with a single read run (TruSeq SR cluster kit v5, Illumina). Unlike the other RNA-seq and ChIP-seq data mined here we have not previously published the SY5Y-MYCN azakenpaullone samples. All RNA-seq samples were performed in biological duplicate and sequenced on an Illumina GAIIx.

ChIP-*seq* was performed on KCN, KCNR and SY5Y-MYCN (un-induced, 24 h and 48 h MYCN induction) cells following the SimpleChIP Plus Enzymatic Chromatin IP Agarose Beads Kit (Cell Signalling Technology) protocol using the MYCN antibody (as above) or anti-Mouse IgG (M5899, Sigma). Libraries were generated using NEXTflex ChIP-Seq Kit (Bioo Scientific) and sequenced as above (single read). Data formatting and peak calling (MACS V1.0.1) were performed using Galaxy (www.usegalaxy.org). Peaks were visualised using SeqMonk v29.0.0 (http://www.bioinformatics.babraham.ac.uk/projects/seqmonk/). We have previously published these ChIP-seq datasets [66]. In this paper we have mined these datasets performing Wnt related analysis. All sequencing samples were performed in biological duplicate and sequenced on an Illumina GAIIx or HiSeq 2500. MYCN's genomic targets were analysed for known regulatory elements using the DiRE [76] tool (http://dire.dcode.org).

Ingenuity pathway analysis (IPA) software was also used for the ITR, pathway and gene ontology (GO) analysis for all omics samples. String (www.string-db.org) was used to generate protein-protein interaction networks.

### Patient microarray data

Sample set composition, sample preparation and generation of single-color gene-expression profiles from primary neuroblastoma were described previously [75]. Raw and normalized microarray data are available in ArrayExpress database (E-TABM-38, E- MTAB-161, E-MTAB-179).

In order to examine the MYCN independent predictive capability of the β-catenin ITR gene signature we also performed correction for MYCN expression for all the genes in this mircroarray dataset. To perform correction for MYCN expression we used LIMMA [102] to fit linear models with MYCN expression levels and MYCN amplification levels as factors. By comparing the fitted coefficients, we estimated the percentage of expression explained by only MYCN expression levels. We then calculated the MYCN corrected expression matrix by subtracting the effects from the MYCN expression level fit, as it is used in LIMMA's batch effect removal function.

### Proteomics

Mass spectrometry based interaction proteomics were conducted on SY5Y-MYCN (un-induced, 4 h, 24 h and 48 h) as previously described [103], for the MYCN protein. MYCN was immunoprecipitated by using Protein A/G PLUS-agarose beads (sc-2003, Santa Cruz) conjugated to MYCN antibody or IgG (as above). We have previously published these datasets [66] and here we have mined them for Wnt related proteins. Three biological and two technical replicates were performed per condition.

### Cell viability and proliferation assay

Cell viability was analysed by MTS assay as described [1], with values normalised to the control of the same day. Measurements for proliferation assays (MTS assay) were taken daily and normalised to the Day 0 (prior to the start of treatment) value. MTS reactions for the proliferation assays were allowed to proceed for the same duration each day. Error bars on all cell viability and proliferation graphs depict standard deviation from the mean.

### High-throughput RNAi screens

SY5Y-MYCN induced and un-induced cells were used for the RNAi screen. We have previously published parts of this RNAi screen [66] and here we have mined the screen for Wnt related genes. Before the high-throughput screening, cell number was titrated to ensure that cell proliferation remained in a linear exponential phase throughout the experiment. The siRNA library targeting human druggable genome (Qiagen GmbH, Germany) consists of four siRNAs per gene. These four siRNAs per gene were pooled into the same well (final assay concentration 50 nM) on 384-well white, clear-bottom assay plates (Greiner Bio-One GmbH, Frickenhausen, Germany), followed by addition of siLentFect (Bio-Rad Laboratories, Hercules, CA) using a Multidrop 384 Microplate Dispenser (Thermo Fisher Scientific Inc, Waltham, MA, USA) for 1 h at room temperature. Cell suspension (1500 cells per well) was thereafter overlaid and the plates were incubated for 72 h at +37°C. Cell viability was measured using CellTiter-Glo Luminescence Assay (Promega, Madison, WI, USA) with Envision Plate-reader (PerkinElmer/Wallac) according to manufacturer's instructions. The raw data were normalized using “loess-samp” normalization method, implemented in R (R Core Team, 2014 http://www.r-project.org/), omitting plate rows 1–2 and 23–24 from normalisation which contain controls, and thereby normalizing the “sample fraction” of the plate, only. After loess correction, z-scores were calculated using robust estimators median and median absolute deviation of the distribution of the corrected values. Hit identification is based on the so called three-sigma rule where data point is considered as a hit if it lays at least 3 standard deviations (SD) from the median of the data distribution. SD was determined applying Huber's Proposal 2 for maximum likelihood estimation. Here, the distribution is winsorized at 1.5 SD before finally estimating the SD. The siRNA pools reducing cell viability by 3 SD from the median of all samples were considered as anti-proliferative hits.

### Zebrafish treatments

Zebrafish (*Danio rerio*, AB and Tg[Fli1:EGFP] strains) were maintained on a 14 h light/10 h dark lighting cycle at 28.5°C. Drug treatments were conducted for 48 h (4hpf - 52hpf) or for 3 h (4hpf - 7hpf). For Wnt activators, treatments for 3 h (4hpf - 7hpf) were also performed and produced axial patterning results comparable with 48 h (4hpf - 52hpf) treatments. Embryos studies were approved by the UCD Animal Research Ethics Committee.

### Accession codes

Sequencing data were deposited in ArrayExpress (www.ebi.ac.uk/arrayexpress) under accession numbers, E-MTAB-2690, E-MTAB-2691, E-MTAB-1684, E-MTAB-2787, E-MTAB-4100 and E-MTAB-2689. The sequence of the mβ-catenin/Tcf3 fusion construct was deposited in Genbank under accession number KM189193.

## CONCLUSIONS

Our findings explain the previously contradictory reports on the role of Wnt signalling in cancer, revealing that Wnt can have both oncogenic and tumour suppressor functions within the same cancer entities. Furthermore, we demonstrate that inhibition and hyper-activation of Wnt signalling are viable therapeutic approaches in their own right, even within the same tumour type. The relevance of the MYCN-β-catenin omics data to neuroblastoma patient outcome was verified both in terms of predicting correlations in expression between genes and by segregating patient outcome. This provides further confidence that our results are applicable to the clinical setting. We revealed not only multiple levels of cross-talk between MYCN and the canonical Wnt signalling pathway but simultaneously provided a predictive gene signature to inform personalised treatment strategies that harness Wnt modulating therapeutics.

## SUPPLEMENTARY MATERIALS TABLES AND FIGURES









## Abbreviations

MNA: MYCN amplified
ITR: inferred transcriptional regulator
hpf: hours post fertilisation
CPMkb: read counts per million adjusted by gene length in kilobases
DE: differentially expressed
CRC: colorectal cancer
IPA: Ingenuity pathway analysis
RA: retinoic acid
FC: fold change
ChIP: chromatin-immunoprecipitation
coIP: co-immunoprecipitation
