# Supplementary material for: Wnt signalling is a bi-directional vulnerability of cancer cells
Source: Oncotarget. 2016 Aug 11;7(37):60310–31. doi: 10.18632/oncotarget.11203 (PMC5312386; doi:10.18632/oncotarget.11203)
Supplement: Supplementary file 1 [file oncotarget-07-60310-s001.pdf]

Wnt signalling is a bi-directional vulnerability of cancer cells

Supplementary Materials

Supplementary Table S1: Opposing expression states (high/low) of selected Wnt signalling genes are associated with better event-free survival

Gene:	Kocak dataset Expression state associated with better event-free survival	*p*-value	SEQC dataset Expression state associated with better event-free survival	*p*-value
APC	high	6.70E-07	high	1.50E-09
WNT3A	high	7.00E-05	high	2.10E-05
WNT5A	high	1.60E-02	high	1.10E-05
FZD10	high	7.70E-10	high	6.20E-07
WNT3	low	1.40E-07	low	3.20E-05
FZD1	low	5.90E-04	low	1.80E-02

Supplementary Table S2 A: Differentially expressed genes which are known β-catenin target genes, and contributed to the prediction of β-catenin functional inhibiton (IPA). See_Supplementary_Table S2A

Supplementary Table S2 B: WNT3A and CTNNB1 ITR activation scores and *p*-values related to Figure 3B, as generated by IPA of RNA-seq data. See_Supplementary_Table S2B

Supplementary Table S3: Differentially expressed genes which are known Wnt3a target genes, and contributed to the prediction of Wnt3a functional inhibiton (IPA). See_Supplementary_Table S3

Supplementary Table S4: Wnt related genes who's DNA was bound by MYCN protein

Gene ID
APC
APCDD1
LRP6
NDP
FZD1
FZD4
FZD5
FZD6
FZD7
FZD8
FZD9
FZD10
WNT2
WNT2B
WNT3
WNT3A
WNT4
WNT5A
WNT7A
WNT8A
WNT9A
WNT10A
WNT11
WNT16
AXIN2
RSPO1
RSPO2
RSPO3
RSPO3
RSPO4
LGR5
LGR6
VANGL1
VANGL2
TCF7L1
TCF7L2
TCF4
TCF21
TCF12
TCF24
CTNNB1
CTNNBL1
LEF1

Supplementary Table S5: WNT3A and CTNNB1 ITR activation scores and *p*-values related to Figure 4B, as generated by IPA from ChIP-seq data

Inferred Transcriptional Regulator (ITR)	*p*-value of overlap
24 h MYCN Overexpression SY5Y-MYCN	
CTNNB1	2.07E-04
WNT3A	4.85E-07
48h MYCN Overexpression SY5Y-MYCN	
CTNNB1	3.10E-06
WNT3A	7.15E-07
KCN	
CTNNB1	5.50E-06
WNT3A	8.32E-06
KCNR	
CTNNB1	8.07E-06
WNT3A	2.00E-04

Supplementary Table S6: ITR DE genes used to generate predictive gene signatures.
See_Supplementary_Table S6

Supplementary Table S7: Neuritogenesis associated diseases or functions annotation (GO terms) data associated with Figure 6C, as generated by IPA

	1 µM Azak IMR32 Activation z-score	*p*-Value	1 µM Azak SY5Y-MYCN- Activation z-score	*p*-Value	SY5Y-MYCN+ Activation z-score	*p*-Value	1 µM Azak SY5Y-MYCN+ Activation z-score	*p*-Value
**outgrowth of neurites**	−2.863	1.02E-08	−2.756	5.60E-05	−3.575	1.57E-11	−4.05	1.63E-15
**f. of PM projections**	−1.388	9.66E-21					−3.743	9.96E-19
**neuritogenesis**	−1.352	1.19E-20	−1.306	−1.306	−2.764	1.43E-11	−3.651	2.61E-18
**morphogenesis of neurons**	−1.227	1.50E-18					−2.249	3.04E-11
**f. of cellular protrusions**	−1.591	2.80E-18	−1.28	2.53E-04	−3.414	6.01E-11	−4.715	4.45E-14
**morphogenesis of neurites**	−1.227	3.30E-18	−0.33	3.17E-05	−1.151	5.30E-08	−2.249	3.53E-11
**branching of neurons**	−1.47	1.09E-13	−0.554	1.35E-04	−1.626	1.47E-06	−2.245	4.43E-10
**shape change of neurons**	−1.288	1.43E-13			−1.457	8.55E-07	−2.121	3.95E-10
**sprouting**	−0.84	2.56E-13	−0.985	9.82E-05	−2.235	3.71E-09	−2.52	2.10E-11
**shape change of neurites**	−1.139	5.48E-13			−1.141	3.44E-06	−1.939	6.59E-09
**branching of neurites**	−1.41	2.45E-12	−0.152	5.52E-04	−1.138	5.17E-06	−2.122	6.03E-09
**axonogenesis**	−0.754	1.85E-10	−1.199	2.81E-04	−1.684	6.01E-06	−3.181	1.34E-07

Supplementary Table S8:TaqMan qPCR assays and SYBR primers

Gene Name	TaqMan Assay ID
MYCN	Hs00232074_m1*
CCND1 (Cyclin D1)	Hs00765553_m1*
DKK1	Hs00183740_m1*
Beta actin (Human ACTB)	4326315E
P0 (RPLP0 [large ribosomal protein])	4310879E
**Gene Name**	**Primer Sequence**
qPCR primers for detection of expression of fusion construct. Product size 173bp.	
mB-cat Fwd 1	AGCTGGCCTGGTTTGATACT
mTcf3 Rev 1	CCTTCTCACTTCGGCGAAAT
Plasmid backbone to detect any plasmid contamination. Product size 76bp.	
Backbone qFwd1	CAACCCGGTAAGACACGACT
Backbone qRev1	GCCTACATACCTCGCTCTGC

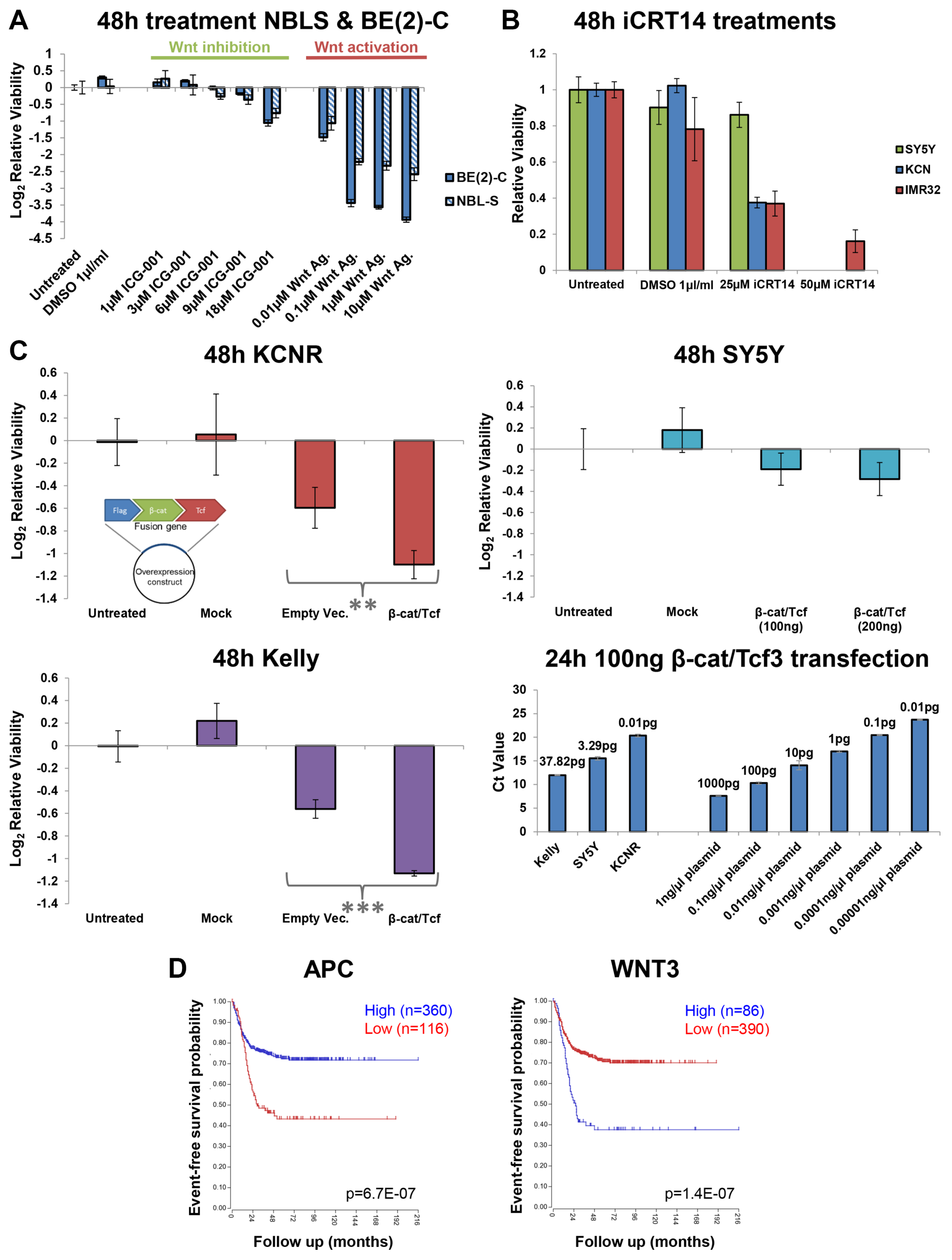

Supplementary Figure S1: Additional cell viability data, upon modulation of Wnt signalling. (**A**) Relative cell viability in NBL-S (MYCN single copy, with somewhat elevated MYCN expression [99]) and BE(2)-C (MYCN-amplified) cells upon Wnt inhibition (ICG-001) and activation (Wnt agonist 1), after 48 h treatments as measured by MTS assay. (**B**) The effect of 48 h Wnt inhibition with iCRT14 on SY5Y, KCN and IMR32 cell viability, assessed by MTS assay with viability relative to control cells. (**C**) *Top two and bottom left panels*: Wnt activation by transfection with constituently active mouse β-catenin/Tcf3 overexpression vector (schematic shown as inset). Relative viability for treatment with no vector (mock), empty vector (empty vec.) or mβ-cat/Tcf3 fusion vector (β-cat/Tcf) at 100 ng (KCNR, SY5Y) or 200 ng (Kelly, SY5Y) per well. Viability was measured by MTS assay and is shown relative to that of untransfected control cells. *Bottom right panel*: The transfection efficiency for each of the cell lines as assed by qPCR of the β-catenin/Tcf3 fusion gene. The PCR product bridges the fusion point between the β-catenin and Tcf3 sequence being specific for the fusion gene. Ct values are graphed with the transfected cell lines on the left and the standard curve generated by using decreasing doses of β-catenin/Tcf3 plasmid, as template, on the left. The pg value of fusion gene per µl of cDNA template is provided above each cell line bar. (**D**) Kaplan Meier event-free survival curves for APC and WNT3 mRNA expression, generated using the Kocak [69] neuroblastoma patient microarray dataset in R2 : Genomics Analysis and Visualization Platform (http://r2.amc.nl).

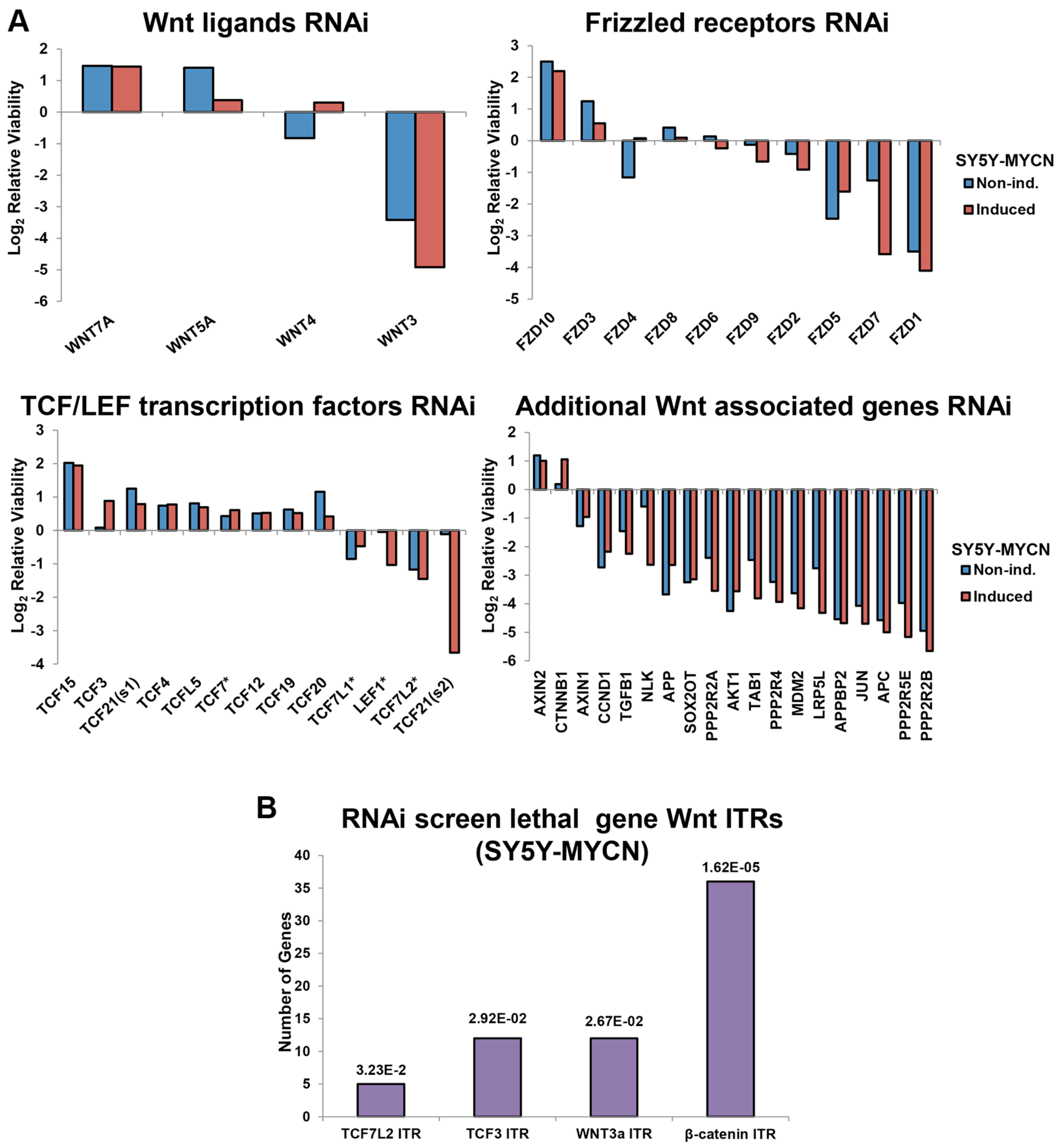

Supplementary Figure S2: Additional Wnt modulation via siRNA data. (**A**) Selected Wnt associated results from a global RNAi screen in induced and un-induced SY5Y-MYCN. The top 3 graphs show all (with mRNA expression above 0.003 CPMkb in SY5Y-MYCN) Wnt ligands, Frizzled receptors and TCF/LEFs transcription factors included in the screen. The bottom graph shows additional selected Wnt associated genes (see Figure 4A schematic) whose knockdown strongly reduced cell viability. Asterisks indicate the 4 primary Wnt associated TCF/LEF genes which were further examined in this study. Where indicated MYCN overexpression was induced 24 h prior to and maintained throughout the subsequent 72 h RNAi treatment. When 2 independent siRNA sets (4 different siRNA's per set) were present for the same gene each set is denoted by (s1) or (s2). (**B**) Numbers of β-catenin, TCF7L2, TCF3 and WNT3A target genes, as identified by IPA ITR analysis, which strongly (greater than 2 standard deviations from the median of the screen) reduced SY5Y-MYCN viability in either condition (MYCN un-induced or overexpressing, overexpression was initiated 24 h prior to and maintained throughout the 72 h RNAi treatment), when knocked down (RNAi screen with druggable-genome siRNA library).The p-value of overlap (between the RNAi hits corresponding to the ITR and all known target genes of that ITR) shown above each bar.

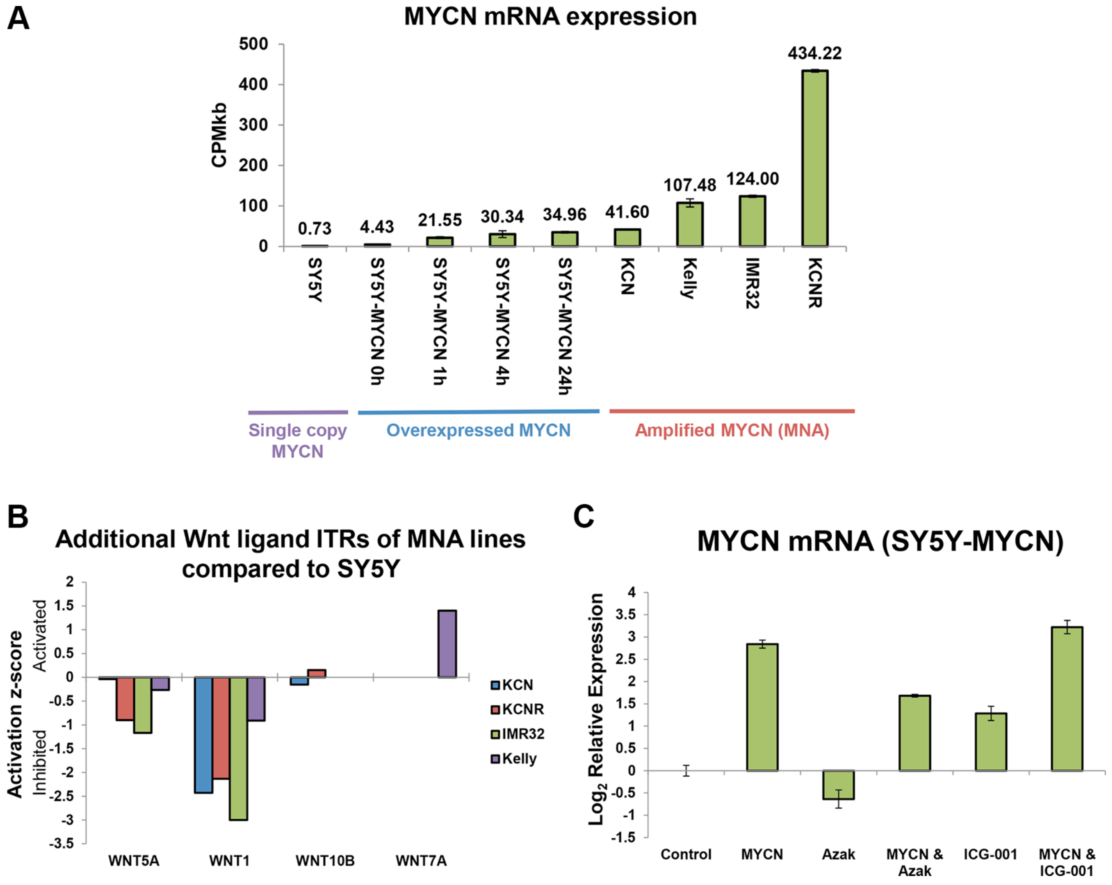

Supplementary Figure S3: Additional Wnt ligands detected as ITRs of the MNA cell lines. (**A**) MYCN mRNA expression across the neuroblastoma cell lines, as quantified by mRNA-seq. CPMkb is read counts per million per kilobase. (**B**) Activation/inhibition z-score plot of WNT5A, WNT1, WNT10B and WNT7A which were ITRs from the comparison of the (**C**) Effect on MYCN mRNA levels of Wnt inhibition (48 h 9 µM ICG-001) or Wnt activation (24 h 1 µM Azaken), in low and high MYCN overexpressing settings.

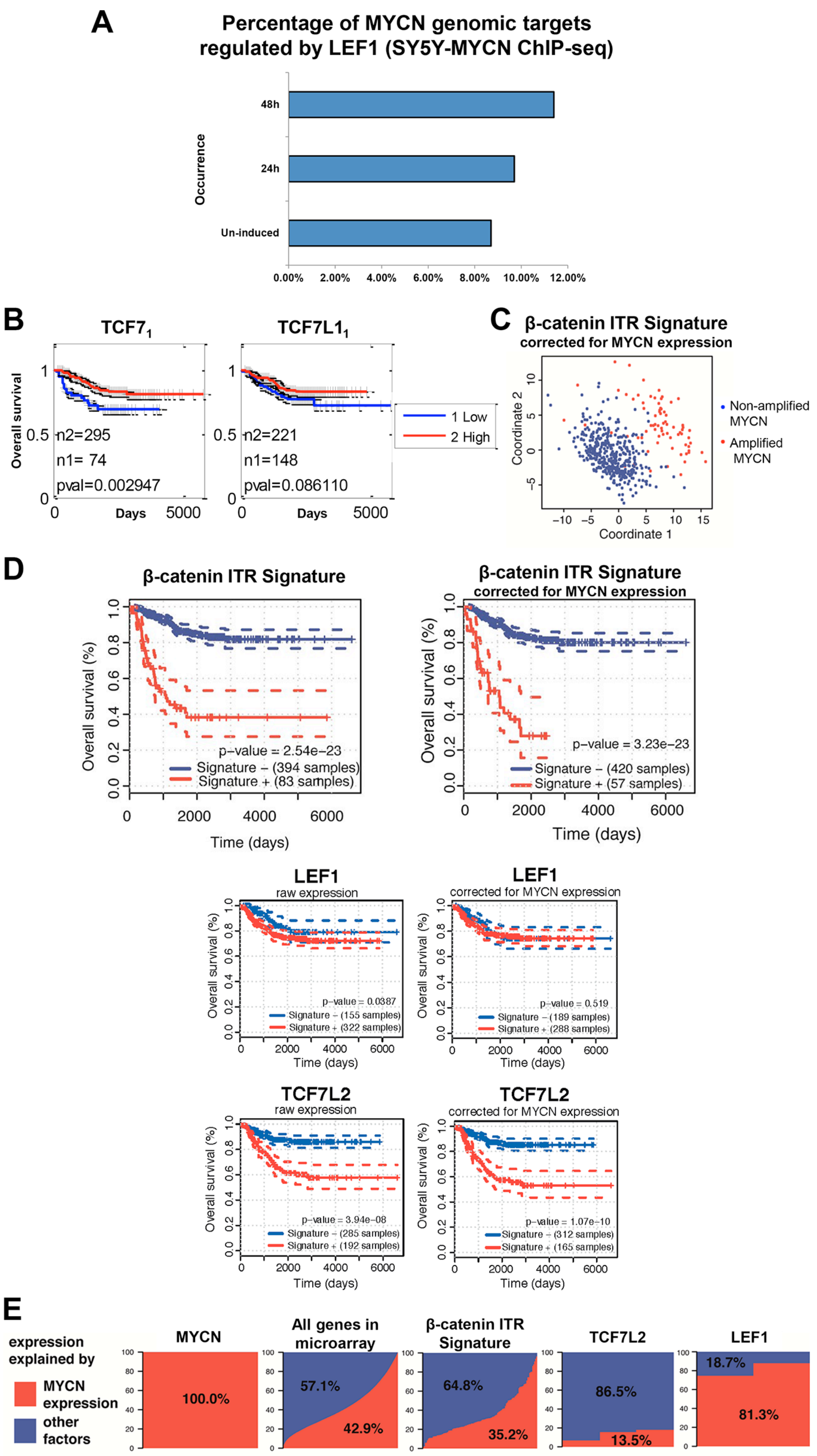

Supplementary Figure S4: Additional TCF/LEF omic and patient data. (**A**) DiRE analysis (http://dire.dcode.org) showing the number of MYCN bound genes (SY5Y-MYCN ChIP-seq) which are also LEF1 targets, i.e. known to have LEF1 binding regulatory elements. The occurrence indicates the fraction genes containing a regulatory element of the particular transcription factor. (**B**) Neuroblastoma patient outcome data segregated by TCF7 and TCF7L1 mRNA expression. Subscript after gene name denotes the specific microarray probe. (**C**) MDS plot of β-catenin ITR signature in the patient microarray dataset. The signature is able to separate MYCN amplified tumours from MYCN non-amplified tumours on the first and second axis of the MDS plot even for the MYCN expression corrected samples. (**D**) Survival curves with 95% confidence intervals for the β-catenin ITR signature for raw gene expression as expressed by the patient microarray (right) and MYCN corrected mRNA levels from the same microarray data (left). (**E**) Percentage of expression explained by MYCN expression levels (red) and other non-MYCN associated factors (blue) as estimated by the LIMMA linear model, see methods section, for MYCN itself (control), all genes present in the patient microarray, the β-catenin ITR signature, TCF7L2 and LEF1.

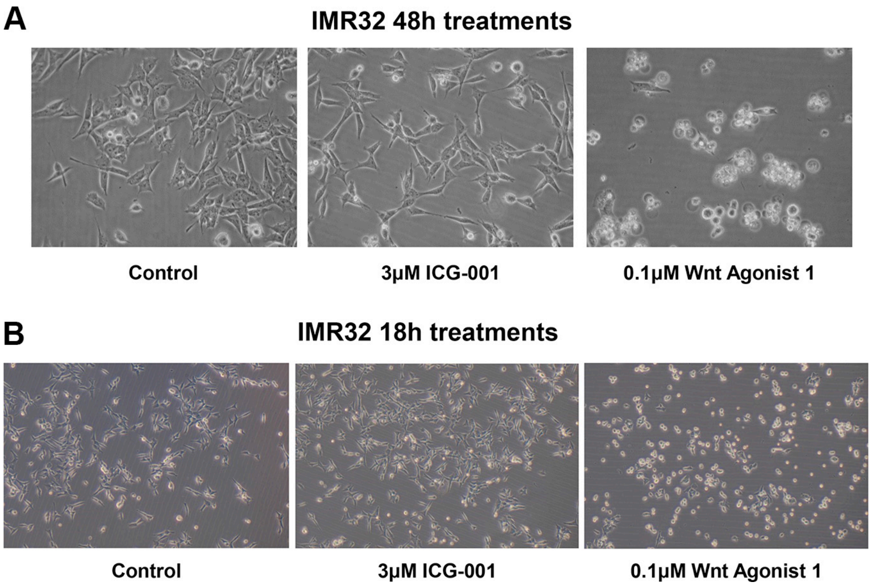

Supplementary Figure S5: Additional imaging on the phenotypic effect of ICG-001 and Wnt agonist 1. (**A**) IMR32 cells treated with ICG-001 and Wnt agonist 1 for 48 h. All panels are imaged at 40× magnification. (**B**) Imaging of IMR32 cells treated for 18 h with Wnt inhibitor, ICG-001 or Wnt activator, Wnt agonist 1. All panels were imaged at 10× magnification.

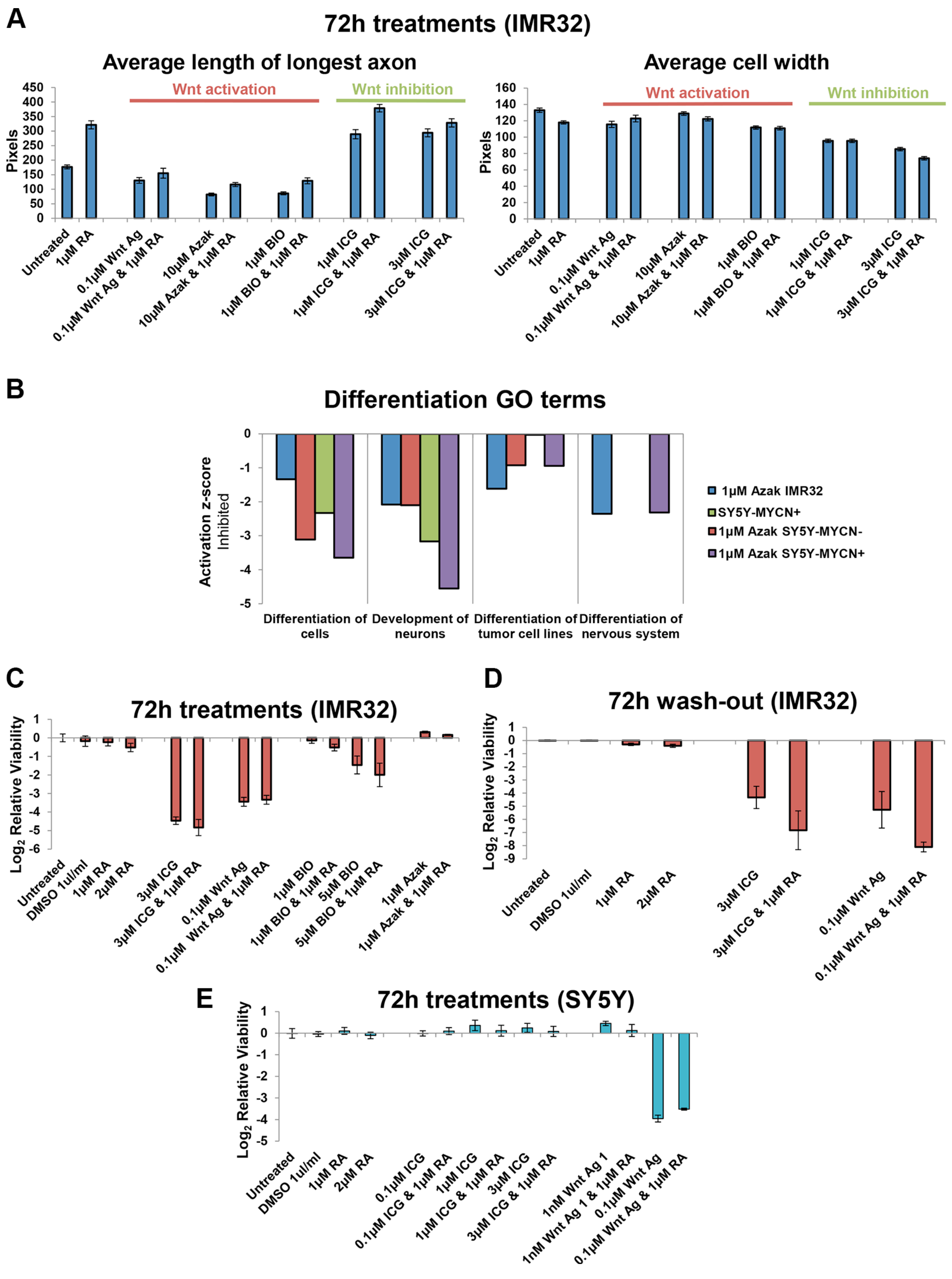

Supplementary Figure S6: Additional differentiation data. (**A**) The average length of the longest axon (top) and cell width (bottom) in IMR32 cells treated for 72 h with individual agents or combination treatments with RA. Range of measured cells (N) per treatment group is 50-171. Error bars depict the standard error of the mean. Measurements made using ImageJ v1.44p (http://imagej.nih.gov/ij). (**B**) IPA disease and function GO term analysis of RNA-seq samples revealed that differentially expressed genes upon 24 h azakenpaullone treatment (in IMR32 or SY5Y-MYCN) or 48 h MYCN overexpression (SY5Y-MYCN), and combination treatment (SY5Y-MYCN) were enriched for differentially expressed genes associated with the inhibition of differentiation. SY5Y-MYCN- denotes un-induced cells, whereas SY5Y-MYCN+ refers to cells in which MYCN overexpression was induced for 48 h. Values are relative to those of the respective controls (IMR32 and SY5Y-MYCN untreated cells). (**C**) Cell viability analysis of the IMR32 MYCN-amplified cell line treated for 72 h with individual agents or combination treatments with RA, as detected by MTS assay. (**D**) IMR32 cells were treated for 72 h (with individual agents or combination treatments with RA), then the small molecules were washed-out and the cells were allowed to recover for a further 72 h before cell viability analysis was performed, using an MTS assay. (**E**) Cell viability analysis of MYCN single-copy SY5Y cells treated for 72 h with individual agents or combination treatments with RA, as detected by MTS assay.

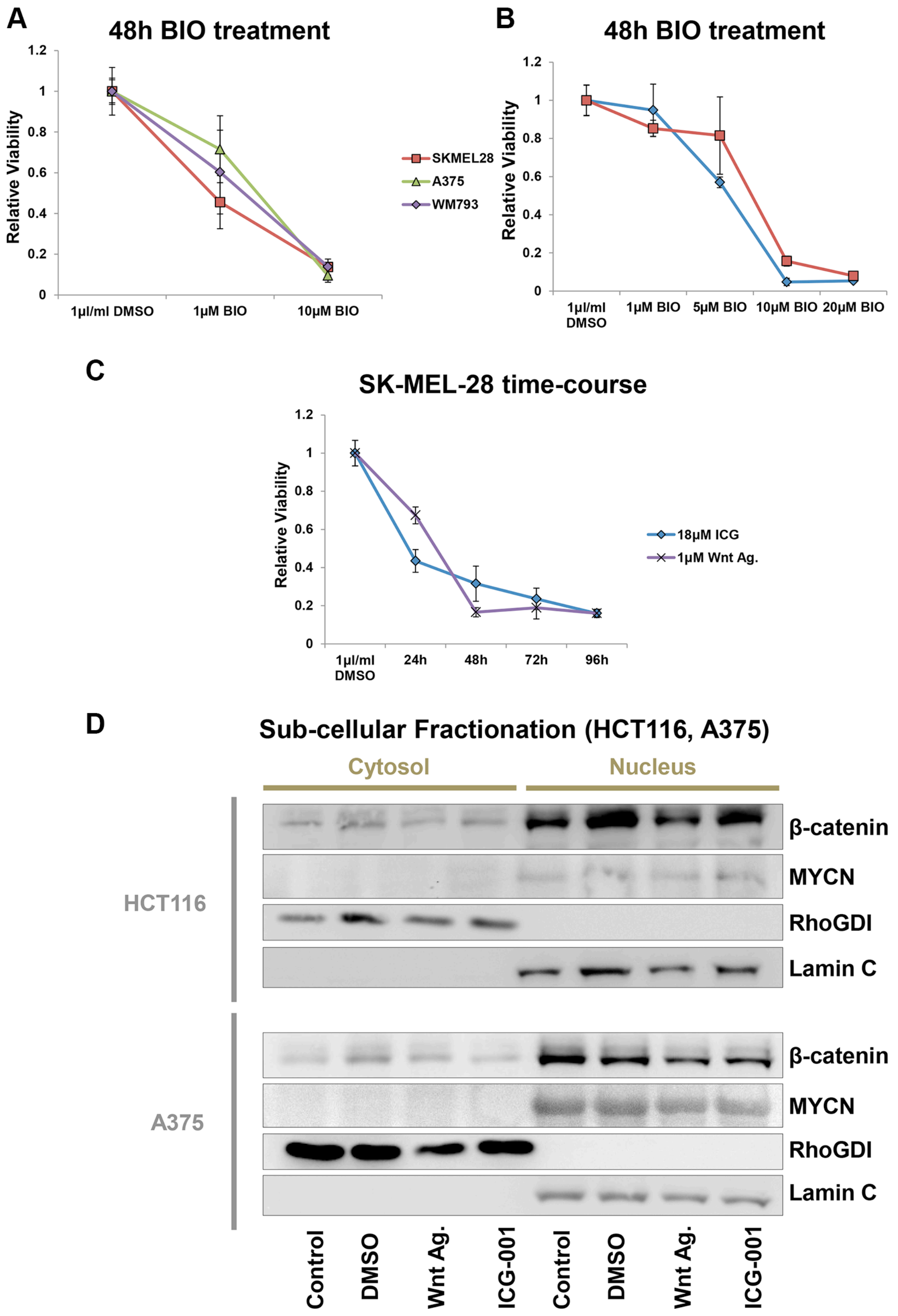

Supplementary Figure S7: Additional Wnt signalling modulation data in malignant melanoma and colorectal cancer cells. (**A**) Cell viability analysis of the dose response of a panel of malignant melanoma cell lines treated for 48 h with BIO (Wnt activation, through GSK3 inhibition), as detected by MTS assay. (**B**) Cell viability analysis of the dose response of colorectal cancer cell lines (HCT116 and HT29) treated for 48 h with BIO (Wnt activation, through GSK3 inhibition), as detected by MTS assay. (**C**) Time-course cell viability analysis of SK-MEL-28 malignant melanoma cell line treated with ICG-001 or Wnt agonist 1 for up to 96 h. (**D**) Sub-cellular fractionation showing nuclear and cytosolic localisation patterns of β-catenin and MYCN proteins in HCT116 and A375 cells. Cystosolic control RhoGDI and nuclear control Lamin C proteins are also shown. Experimental conditions were untreated control cells, or 3 h 1 µl/ml DMSO vehicle control, 1 µM Wnt Agonist 1 or 18 µM ICG-001 treated cells.
